# Nanoarchitectonics of Porous Carbons Templated by Inorganic Metal Oxides and Alkali Metal Salts for Energy and Environmental Applications

**DOI:** 10.1002/advs.202517776

**Published:** 2026-03-26

**Authors:** Gurwinder Singh, Jibi Kunjumon, Harleen Kaur, Narinder Singh, Gregory Franklin, Arpita Pandey Tiwari, Lukas Van Zweiten, John Kennedy, Ajayan Vinu

**Affiliations:** ^1^ Global Innovative Centre for Advanced Nanomaterials (GICAN) College of Science Engineering and Environment (CESE) School of Engineering University of Newcastle Callaghan NSW 2308 Australia; ^2^ Department of Chemistry Indian Institute of Technology Ropar Rupnagar Punjab 140001 India; ^3^ Institute' of Plant Genetics Polish Academy of Sciences Strzeszyńska 34 Poznan 60‐479 Poland; ^4^ Department of Stem Cell & Regenerative Medicine and Medical Biotechnology Centre for Interdisciplinary Research D. Y. Patil Education Society (Deemed to be University) Kolhapur Maharashtra 416006 India; ^5^ NSW Department of Primary Industries Wollongbar Primary Industries Institute Wollongbar NSW 2477 Australia; ^6^ National Isotope Centre GNS Science PO Box 31312 Lower Hutt 5010 New Zealand

**Keywords:** metal oxide templates, metal salts templates, multipurpose applications, porous carbons, templating

## Abstract

Inorganic nanoparticles are widely used as sacrificial templates for the synthesis of porous carbons due to their good thermal stability, characteristic shapes, tunable sizes, compatibility with carbon precursors, and lower cost and toxicity than conventional silica‐based templates. Their use not only ensures the development of hierarchical porosity but also the creation of short‐range graphitic domains in the carbon matrix. These qualities make porous carbons suitable for different applications, including adsorption, separation, catalysis, and energy storage and conversion. Within the series of inorganic nanoparticle templates, metal oxides such as MgO, ZnO, Fe_2_O_3_, Fe_3_O_4_, and MnO_2_, and alkali metal salts such as NaCl and KCl stand tall as templates. They are thermally and structurally stable, do not react with the carbon precursor, and do not require high‐cost, harsh removal methods such as HF washing. This review provides up‐to‐date discussions of metal oxides (MgO, ZnO, Fe_2_O_3_, Fe_3_O_4_, and MnO_2_) and metal salts (NaCl, KCl, and composite salts) to produce porous carbons and addresses other aspects of their structures. This is a focused review that critically analyzes the recently published literature and will serve as a guiding framework for the future design and development of porous carbons.

## Graphical abstract

This review explores inorganic metal oxides and metal salt nanoparticles templated porous carbons, highlighting their synthesis, structural features, and performance in energy and environmental applications. It critically compares template types, porosity control, and functional outcomes across recent literature.

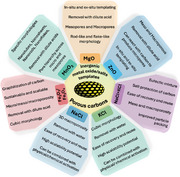

## 1 Introduction

Porous carbons have proven to be versatile materials with immense potential in various application fields, including energy and environment.^[^ ^1^, ^2^, ^3^ ^]^ Their performance in different applications relies mainly on their porosity domains, including surface area, pore volume, pore size, and surface chemistry.^[^ ^4^, ^5^, ^6^ ^]^ They can be synthesized using various strategies, the most common and cost‐effective being the carbonization of carbon‐containing precursors, followed by subsequent activation by utilizing either physical or chemical means.^[^ ^7^ ^]^ The activation approach imparts characteristic properties to the synthesized porous carbons. In general, the pore domains can be precisely controlled in various dimensions, including micro, meso, or macro, and the surface chemistry can be constructed with the desired functionalization. However, one of the significant drawbacks of porous carbons prepared by these conventional methods is the lower control over their pore size distribution, ranging from a few nanometres to several nanometres.^[^ ^8^, ^9^, ^10^, ^11^, ^12^ ^]^ This often limits their performance in applications such as gas storage, energy storage, and catalysis. To specifically engineer porous carbons with a desired porous architecture, including a narrow pore size distribution and surface chemistry, techniques such as templating have often been utilized.^[^ ^13^ ^]^ Templating not only allows precise control of the pore structure but is also a highly reproducible technique. However, the major downside of using conventional templates, such as silica, is the high expense of the process due to the high cost of templates and their harsh removal methods using non‐environmentally friendly acids such as hydrofluoric acid (HF).^[^ ^14^ ^]^

During templating, the synthesized porous carbon mimics the inverse replica of the template, which is sacrificed when the template is removed. For hard templating, silica, zeolites, or metal oxides are generally employed as templates, while block copolymers or organic micelles are used for soft templating.^[^ ^15^ ^]^ The well‐known silica‐templating approach can be used to generate pores in carbon and other materials. Still, as mentioned above, its lack of environmental friendliness is always an obstacle to large‐scale commercialization, as harsh acidic conditions are required to remove the templates.^[^ ^16^ ^]^ In recent years, the production of templates based on inorganic nanoparticles has attracted considerable attention.^[^ ^17^ ^]^ These offer two advantages: they can be easily removed with dilute acids or water, and the material's pore structure can be customized as required by varying the template source from a vast library. For instance, zinc oxide (ZnO) can be used as a hard inorganic template to produce hierarchical porous carbon, with a large surface area, while simultaneously creating local graphitic domains in the structure.^[^ ^18^ ^]^ The presence of ZnO creates a space‐confinement effect, and its reaction with carbon forms pores in the carbon structure by driving the escape of volatiles, such as carbon monoxide (CO). In addition, the presence of Zn also promotes the catalytic graphitization of porous carbon. Each inorganic template behaves differently during pore structure formation. For instance, while ZnO can create a hierarchical porosity consisting of a majority of mesopores and fewer macroporous domains, magnesium oxide (MgO) can form a large number of mesopores and macropores, but only a negligible amount of micropores due to its action as a spacer, as it does not chemically react with carbon.^[^ ^19^, ^20^, ^21^ ^]^ On the other hand, iron oxide (Fe_2_O_3_) can form micro‐ and mesopores, accompanied by hollow carbon structures.^[^ ^22^ ^]^ In contrast, manganese oxide (MnO_2_) can create a majority of mesopores and a small number of micropores, along with the creation of the nanosheet‐type structures.^[^ ^23^ ^]^ Accordingly, the resultant porous carbons become suitable for specific applications, including energy storage and conversion, and adsorption and separation. For example, MgO templated carbons are well suited for supercapacitors as they enable faster ion transport in the mesopores,^[^ ^24^ ^]^ ZnO templated carbons are effectively suited for gas storage^[^ ^25^ ^]^ and oxygen reduction reaction (ORR),^[^ ^26^ ^]^ MnO_2_ templated carbons perform well in both supercapacitors and ORR.^[^ ^27^ ^]^ In contrast, Fe_2_O_3_ templated carbons show high CO_2_ adsorption capacity.^[^ ^28^ ^]^ MgO is widely utilized as an inorganic metal oxide for introducing a templating effect in porous carbons owing to its abundance and low cost, high stability, easy removal due to the weak interaction with the carbon precursors, versatility to tailor the pore structures, and the ability to offer a high yield of the final product.^[^ ^29^ ^]^ There are many research articles on the use of MgO as a template for the development of porous carbon materials.^[^ ^30^, ^31^ ^]^ The MgO template can be obtained from a variety of salts, which can be combined either “in situ” or “ex situ” to create a templating effect in porous carbons, derived from various carbon feedstocks.^[^ ^32^, ^33^ ^]^ The magnesium salts, which ideally decompose in a temperature range of 300–800 °C, can yield MgO nanoparticles. Various magnesium salts, such as magnesium nitrate, magnesium chloride, magnesium acetate, magnesium oxalate, magnesium carbonate, magnesium hydroxide, magnesium citrate, magnesium tartrate, magnesium gluconate, and magnesium lactate, can serve as effective sources of the MgO templating effect. However, not all of them have been tested for MgO's templating effect.

In addition to the inorganic metal oxides, the inorganic alkali metal salts, such as sodium chloride (NaCl)^[^ ^34^ ^]^ and potassium chloride (KCl),^[^ ^35^ ^]^ are also remarkable templates for porous carbons. Of these two, sodium chloride offers an exciting prospect for the development of porous carbons, primarily in the macroporous structural domains, due to its templating effect centered on the cubic structure.^[^ ^36^ ^]^ Carbonization of precursors in the presence of NaCl can also generate a reasonable amount of micro and mesopores, making the developed porous carbons useful for a myriad of applications.^[^ ^37^ ^]^ The development of short‐range graphitic domains in the porous carbon matrix is another feature of employing NaCl as an inorganic template. From an environmental perspective, any residual NaCl can be washed off with water and recovered with 80% efficiency. The only disadvantage of NaCl templated carbons is the low surface area, which can be compensated for by preserving a higher content of heteroatoms in the carbon matrix due to the mild chemical action of NaCl. In addition, NaCl‐templated carbons have sufficient carbon content, enabling further chemical activation with agents such as potassium hydroxide (KOH). Overall, NaCl provides an economical and environmentally friendly route to producing porous carbons with the properties needed for various applications. KCl behaves like NaCl in terms of its properties, i.e., crystal size, lattice, and melting points are not far apart. The larger size of potassium should lead to the formation of larger voids in porous carbons. Both NaCl and KCl also serve as effective dual templates for creating meso and macroporosity in porous carbons. These salts have also been investigated in conjunction with chemical activating agents such as KOH and zinc chloride (ZnCl_2_) to increase the influence of porosity in porous carbons.^[^ ^38^, ^39^ ^]^

**Figure 1:**
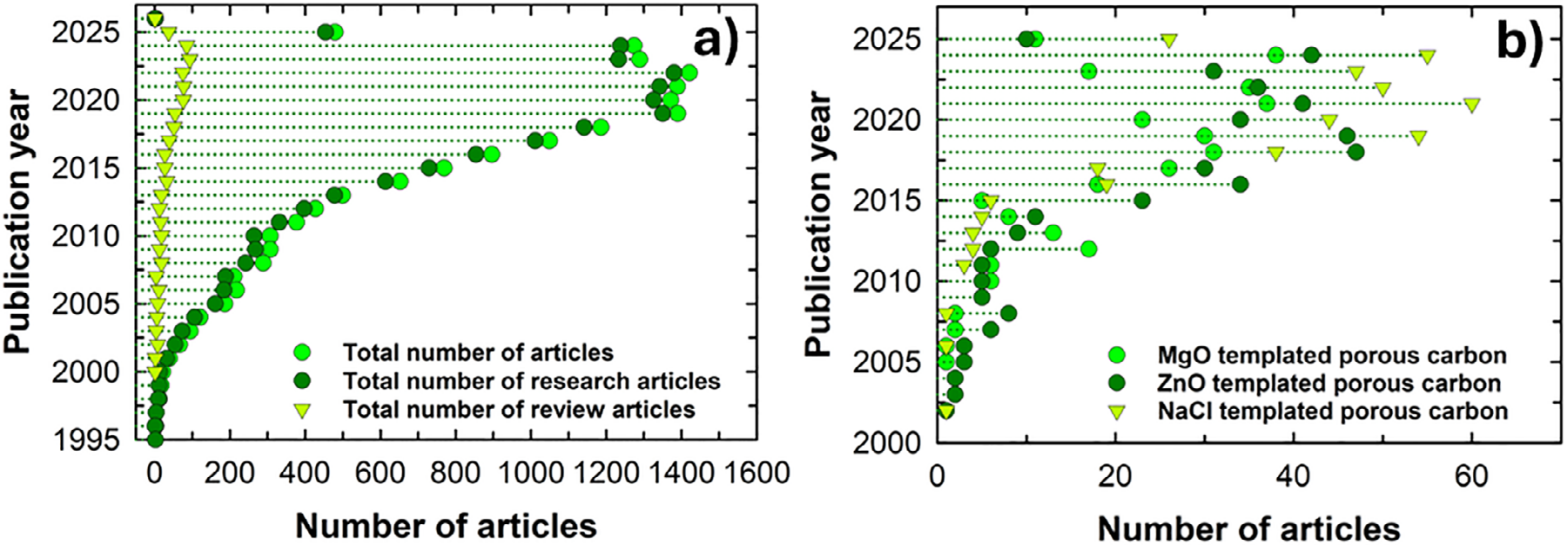
Statistics on the number of publications versus publication year on the topics of a) “templated porous carbons”, and b) some prominent inorganic templates including MgO, ZnO, and NaCl (data extracted from Web of Science on 07/08/2025).

A vast library of data is available when searching for the term “templated porous carbons” in the Web of Science database, with research articles outnumbering review articles (**Figure** **1**a). This database potentially covers all types of templating processes, regardless of whether the templated material is silica or non‐silica, and it yielded a total of 16368 articles (predominantly research articles) published in the last three decades. Narrowing the search to a few particularly prominent inorganic templates, such as MgO, ZnO, and NaCl (using the standard command MgO/ZnO/NaCl templated porous carbon in the Web of Science), shows that such research only picked up pace a decade ago and continues with 332, 440, and 436 published articles, respectively (Figure 1b). This indicates that inorganic templates for fabricating advanced porous carbon‐based materials, which can be utilized in various applications, are garnering increasing attention. In recent review articles, Zhang et al. (2021) reviewed hard, soft, and self‐templates for supercapacitors,^[^ ^40^ ^]^ followed by a similar article in 2022 by Wang et al.^[^ ^41^ ^]^ Recently, Díez et al. reviewed inorganic templates together with polymeric materials,^[^ ^42^ ^]^ and later Pavlenko et al. (2022) reviewed organic and metal oxides, silica, polymers, metal‐organic frameworks (MOFs), and materials of biological origin for various applications.^[^ ^43^ ^]^ This review is unique in that it focuses exclusively on inorganic templates and covers two areas: inorganic metal oxides and inorganic alkali metal salts. Each area has been discussed based on the adopted synthesis attributes, characteristics, and properties of the materials — particularly porosity (surface area, pore volume, and pore size) — as well as application statistics to demonstrate their broad adaptability. Wherever possible, comparative insights are presented to better understand the capabilities of various inorganic templates to produce porous carbons with interconnected pores. In addition, the multi‐salt/oxide templating approach is also briefly addressed, and comments are made on future work.

## 2 Synthesis and Application Perspectives of Inorganic Metal Oxide Nanoparticle Templates

**Figure 2:**
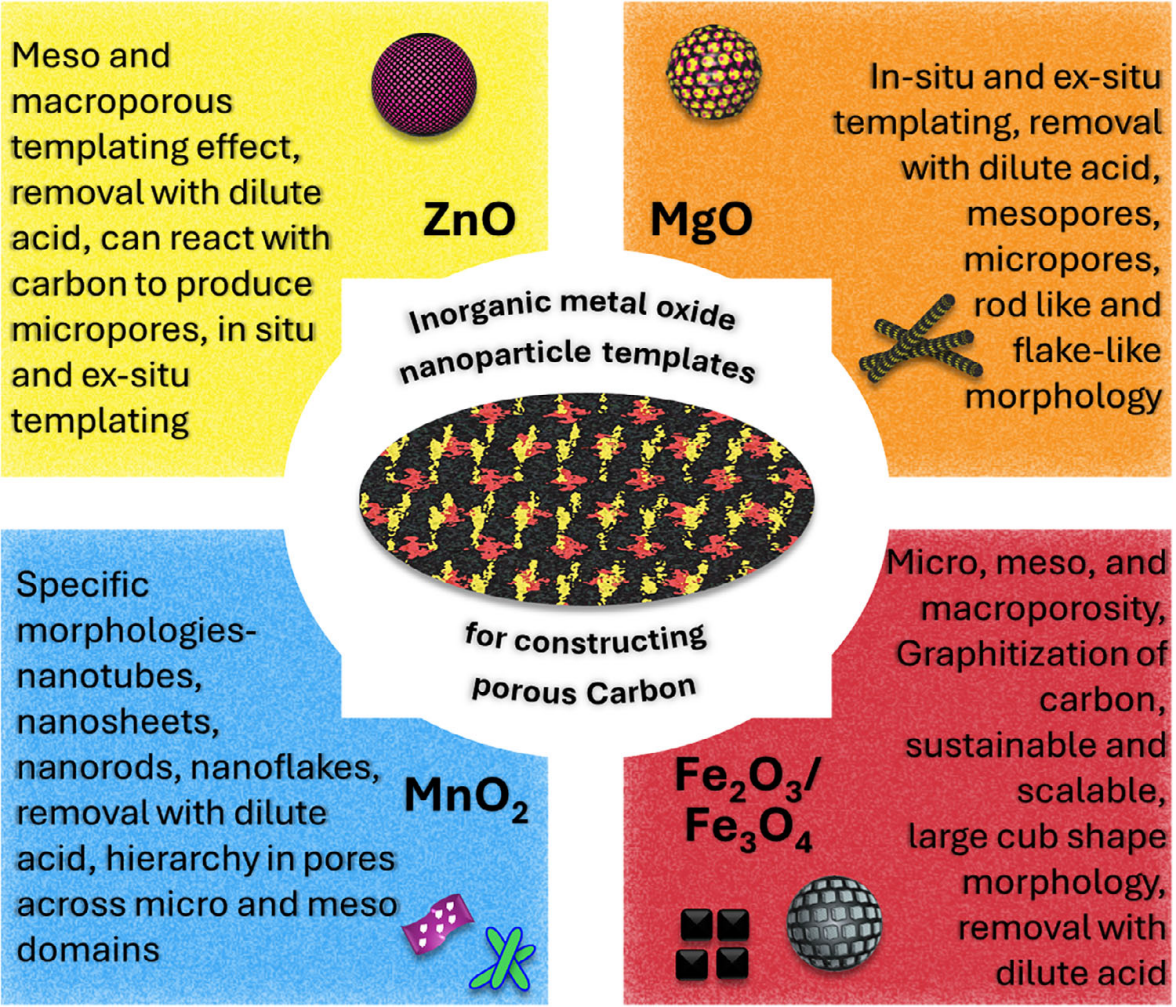
Various aspects of inorganic metal oxide nanoparticles as templates for synthesizing porous carbons.

As described in the introduction, inorganic metal oxide nanoparticles offer several advantages for templating the synthesis of porous carbons. The salient features of MgO, ZnO, Fe_2_O_3_/Fe_3_O_4_, and MnO_2_ are represented in **Figure** **2**, and a summary of various properties of porous carbons prepared via a hard inorganic metal oxide templating method is provided in **Table** **1**.

**Table 1:** Summary of the various physico‐chemical features of porous carbon obtained using the templating action of inorganic metal oxide nanoparticles.

Material	Template/s and carbon precursor/s	Template removal technique	Mechanism of templating/properties of porous carbon	SA [m^2^ g^−1^], PV [cm^3^ g^−1^] and other features	Application attributes	Refs.
**MgO**						
NHPCs	MgO/ Polyacrylamide	Acid and water washings	MgO template and carbon precursor are combined by electrostatic self‐assembly, 3D interconnected hierarchical porous structure, large specific surface area, and a large amount of heteroatoms, nitrogen (10.97 at% %) and oxygen (10.3 at% %)	989.836 m^2^ g^−1^ and 1.052 cm^3^ g^−1^ for NHPC‐800, amorphous carbon materials containing C, N, and O, and N occur as pyridinic N, pyrrolic N, quaternary N, and oxidized N	Specific capacitance: 295.3 F g^−1^ at 0.5 A g^−1^, 100.76% capacitance retention at 10000 cycles in 6M KOH, symmetrical supercapacitor yields high energy density of 15.16 Wh kg^−1^ in 1 M Na_2_SO_4_ aqueous electrolyte.	[32]
MS‐OMCT/ S‐OMCT	SiO_2_@MgO/ Phenolic resin	A mixed acid solution of HCl and HF‐based washing	Rich pseudocapacitive oxygen species‐enriched ordered mesoporous carbon with a large surface area and partial graphitization of carbon structure delivers a synergistic effect of both electrochemical double‐layered capacitive (EDLC) and pseudocapacitive behaviour in 1 M H_2_SO_4_	1175 m^2^ g^−1^ and 2.55 cm^3^ g^−1^ for MS‐OMC1073, amorphous carbon materials containing C, H and O	Specific capacitance: 257 Fg^−1^ at 0.5 A g^−1^, capacitance retention:77.8% at higher current densities	[52]
H‐NOCBs	MgCl_2_ / PDA	Acid wash with 1M HCl	The MgO template created at 600 °C replicates its hollow cubic shape morphology onto porous carbon, generating a reasonable surface area of ≈410 m^2^ g^−1^ spread across micro and mesoporous domains.	410.1 m^2^ g^−1^, 3.93 nm /presence of both mesopores and micropores	Cycling stability with the retained capacities of 178.8 mA h g^−1^ (500 mA g^−1^) and 123.3 mA h g^−1^ (1000 mA g^−1^) after 2500 and 10000 cycles, respectively.	[53]
TC	4MgCO_3_.Mg(OH)_2_.4H_2_O /CO_2_	No removal/template recycled via a green route	The MgO template created at 800 °C helps in forming a templated carbon structure with a flake‐like morphology, creating high surface area, mesoporosity, and controlled porosity	>1000 m^2^ g^−1^, electronic conductivity >300 S m^−1^, graphitized carbon, and high recyclability	In (KOH/H_2_O, >154 F g^−1^), in organic (Et_4_NBF_4_/AN, >140 F g^−1^) and ionic liquid (EMIMBF_4_, >178 F g^−1^) electrolytes, and ≈100% CE	[54]
HPC	4MgCO_3_.Mg(OH_2_).5H_2_O/ K_3_C_6_H_5_O_7_.H_2_O	Acid wash using dilute HCl	MgO template imparts micro, meso, and macroporosity to carbon, which forms into carbon chains, rings, and graphene fragments	1796 m^2^ g^−1^, 2.73 cm^3^ g^−1^/ I_D_/I_G_ value for HPC‐2 is 1.03	Specific capacitance of 305.5 F g^−1^/0.5 A g^−1^ and 173.1 F g^−1^/50 A g^−1^ for a three‐electrode system	[55]
CPC‐M	Magnesium citrate/ Coal	Acid wash with dilute HCl	“in situ” MgO templating induced porosity, catalytic graphitization, and high surface area	1149 m^2^ g^−1^/0.63 cm^3^ g^−1^ possesses a high specific surface area, graphitized structure, and enhanced biocompatibility	Microbial fuel cell Chemical Oxygen Demand removal rate of 46.1 %	[61]
HC600:1500	Magnesium gluconate/ Glucose	Acid wash with dilute HCl	The MgO template created at 600 °C causes the formation of nano‐sized pores at 1500 °C, along with thin graphitic layers	839 m^2^ g^−1^, less than 2 nm/ Large total volume of the nanopores (or nano‐sized voids) and surrounding thin graphitic layers with a wide interlayer space	Aprotic sodium cell specific capacity of 478 mAh g^−1^, Coulombic efficiency of 88 %	[62]
MC800	Magnesium citrate/ Magnesium citrate	Acid wash with 1M HCl	Magnesium citrate forms MgO at high temperatures, which shapes the porous structure of carbon derived from citrate	1673 m^2^ g^−1^/3.91 nm/ High surface area with abundant small mesopores	Lithium‐ion capacitor Energy density of 152.2 Wh kg^−1^ and power density of 14.3 kW kg^−1^	[63]
HC‐Mg‐12.5%	Magnesium acetate/ starch	Acid wash with 1M HCl	MgO template created open pores in the carbon, which were then turned into closed pores at 1500 °C	3.92 m^2^ g^−1^/0.15 cm^3^ g^−1^ (closed pore)/Pseudo graphitization and disordered carbon structure were observed	Reversible capacity of 369.17 mAh g^−1^ and an initial coulombic efficiency of 84.68% were obtained for a sodium ion battery	[64]
**Fe_2_O_3_/Fe_3_O_4_**						
HPCFs/ NDHPCFs	Fe_2_O_3/_ Resol	Creating cubical macroporous structures/	Hard templating using Fe_2_O_3_ cubes, NaOH activation, and urea incorporation produced materials with macro and micro pores and nitrogen doping.	1100 m^2^ g^−1^/0.73 cm^3^g/ Pore enlargement was observed after nitrogen doping	0.82 mmol g^−1^ at 25 °C and 20 bar.	[28]
**MnO_2_**						
SnO_2_/C	KMnO_4_/ PDA	Acid wash with 0.5M C_2_H_2_O_4_	MnO_2_/SnO_2_ nanocables generated via HTC were coated with PDA and carbonized to remove MnO_2_. MnO_2_ leaves a mesoporous effect and 1D hollow nanostructures behind in SnO_2_/C nanotubes	270 m^2^ g^−1^/ Mesoporous structure	Reversible lithium storage capacity of 596 mA h g^−1^ after 20 cycles	[81]
PDHC	KMnO_4_/PANI	Reduced with acid and washed with water	MnO_2_ nanotubes assist in the polymerization of aniline into nanotubes and self‐sacrifice into Mn^2^ and MnO_2,_ imparting mesoporosity to PDHC along with a high surface area	2040 m^2^ g^−1^/1.15 cm^3^ g^−1^ / Abundant surface functional groups, excellent chemical stability, and controllability	Specific capacitance of 467 F g^−1^/1 A g^−1^ in a 3‐electrode system, good activity for ORR	[27]
S&N‐CNS	MnO_2_ nanosheets/ Pyrrole	Centrifugation with deionized water and ethanol multiple times	Templates help in enhancing the reaction dynamics, active/defect sites, and ion‐transport kinetics /Carbon precursor has low cost and good physicochemical stability	The C‐S bond helps in improving the electrochemical capacity and cycling longevity	Specific capacity of 433.9/523.7 mAh/g^−1^ at 0.1/0.2 A g^−1^ and a stable cycling life over 2000/3000 cycles at 5.0 A g^−1^ for K+/Na+ storage. Potassium ion hybrid capacitor gives an energy density of 124.0 Wh kg^−1^ at a power density of 165.3 W kg^−1^.	[23]
N/O‐HCNTB	MnO_2_ nanowires/ Citric acid	Rinsed in aqueous citric acid solution for 12 h	Increase specific surface area, provide active sites for ion transport, enhance ion/electron diffusion kinetics, and buffer volume changes, improving structural stability and capacity retention	154.9 m^2^ g^−1^/0.24 cm^3^ g^−1^/ Higher carbonization temperature improves the structural order of the material and confirms the presence of micro and mesopores.	Delivers an initial charge capacity of 427 mAh g^−1^ and maintains 94.8% capacity retention after 200 cycles at 100 mA g^−1^.	[82]
**ZnO**						
HPC	Commercial ZnO/ Petroleum pitch	Acid wash with 3 M HCl under stirring for 2 hr	ZnO creates 20–60 nm holes in the carbon structure. Lower concentration leads to meso, and higher concentration leads to a mixture of meso and macropores	1469 m^2^ g^−1^/1.10 cm^3^ g^−1^, a tap density of 0.31 cm^3^ g^−1^, and a hierarchical structure containing micro, meso, and macropores	Electrosorption capacity of 9.94 mg g^−1^ in 10.0 min with a maximum electrosorption capacity of 10.62 mg g^−1^ at 1.2 V in a 5.0 mM NaCl solution	[99]
WCS biochar/ZnO/KOH	Commercial ZnO/ Water Caltrop shell	Water wash and then acid wash with 37% HCl solution	Multiporosity spanning across micro, meso, and macropores was achieved by varying the weight ratio of ZnO. ZnO produces a space confinement effect	1537 m^2^ g^−1^/0.89 cm^3^ g^−1^, pore size of ≈20 nm was achieved, typical D and G bands for amorphous carbon were observed in Raman spectra	Specific capacitance of 128 F g^−1^ at 5 mV s^−1^ with a low ohmic resistance in 1 M LiClO_4_/PC electrolyte	[100]
PCSMs	In situ ZnO generated from thermal decomposition of Zn_5_(OH)_6_(CO_3_)_2_/ Coal tar pitch	Acid wash with 1 M HCl and repeated washings with water	ZnO templating caused the formation of a high surface area and mesopores, which were further widened with KOH activation	1359.88 m^2^ g^−1^/2.67 cm^3^ g^−1^/unique microspherical morphology was achieved, composed of several carbon nanosheets	Specific capacitance of 313 F g^−1^ at 1 A g^−1^ and high‐rate capability of 81.9% capacitance retention at 50 A g^−1^ in a three‐electrode system.	[101]
CoO@Fe‐NCZnO	ZnO/Fe/ Fe_3_O_4_/ Polyacrylamide	Washed with deionized water 3 – 4 times	“in situ” polymerization, Fe incorporation, pyrolysis, acid etching, and hydrothermal deposition of CoO using ZnO and FeCl_3_ as templates facilitated a hierarchical porous structure with FeN_x_ active sites	989.836 m^2^ g^−1^, 1.052 cm^3^ g^−1^ / 3D interconnected hierarchical porous structure, large SA and functional groups (N and O)	ORR/OER Half‐wave potential (E1/2) of 0.917 V (vs. RHE) and a low potential of 1.556 V (vs. RHE) at a current density of 10 mA cm^−2^	[71]

**SA** – Surface area, **PV**‐ Pore volume; **NHPCs**‐N‐doped hierarchical porous carbon made from polyacrylamide and MgO template; **MS‐OMCT** – Ordered mesoporous carbons templated by as‐made SiO_2_@MgO nanoparticles; **H‐NOCBs** – N/O dual‐doped carbon boxes; TC‐ **HPC** – Hierarchical porous carbon, **CPC‐M –** Coal‐based porous carbon catalytic materials using magnesium oxide as a templating agent and attached to a carbon cloth; **HC600:1500 –** Hard Carbon pre‐treatment at 600 °C and post‐treatment at 1500 °C; **MC800 –** MgO templated porous carbon synthesized from magnesium citrate at 800 °C; **HC‐Mg‐12.5%** – Hierarchical carbon synthesized from starch, **HPCFs/NDHPCFs**‐ Hierarchically porous carbon frameworks and their nitrogen‐doped variants; **SnO_2_/C**‐ Tin oxide and carbon nanotubes nanocomposite; **PDHC** – N‐doped hierarchically porous carbon derived from polyaniline and MnO_2_ as sacrificial template; **S&N‐CNS –** Sulfur and nitrogen doped carbon nano sheets synthesized using MnO_2_ nanosheets template subjected to heat treatment at 600 °C for 2 hrs: **N/O‐HCNTB** – N/O‐co‐doped 1D mesoporous hard carbon nanotube bundles template with MnO_2_ nanowires heated at 900 °C for 2 hrs; **WCS biochar/ZnO/KOH** – Porous carbon obtained from water caltrop shells via ZnO templating and KOH activation; **PCSMs** – Porous carbon sheet microspheres synthesized via ZnO templating and KOH activation; **CoO@Fe‐NCZnO** – hierarchical porous carbon‐based catalyst loaded by single‐atomic FeN_x_ and CoO nanoparticles synthesized using ZnO, Fe, and Fe_3_O_4_ as nano templates; **PDA**‐polydopamine, **C_2_H_2_O_4_** – Oxalic acid, **PANI** – Polyaniline, **TC** – Template Carbon, **HPC** – Hierarchical porous carbon, **4MgCO_3_.Mg(OH_2_).5H_2_O** – Basic magnesium carbonate, **K_3_C_6_H_5_O_7_.H_2_O** – Potassium citrate; Applications‐ **ORR**‐Oxygen reduction reaction, **OER** = Oxygen evolution reaction, **EDLC** – Electric Double layer Capacitance, **F g^−1^** – Faraday per gram, **A g^−1^** – Ampere per gram, **mmolg^−1^** = millimole per gram

### 2.1 MgO

MgO stands out among inorganic oxide‐based templates for its use in creating templated porous carbons.^[^ ^44^ ^]^ Compared to microporous templates such as zeolites and silicas, MgO generally produces a mixture of microporous and mesoporous domains in the porous carbons.^[^ ^45^ ^]^ Since templated porous carbons are often synthesized at high temperatures (≈800 °C), the good thermal stability of MgO is a significant factor contributing to its behavior as a rigid sacrificial template.^[^ ^46^ ^]^ It melts and boils at extremely high temperatures of 2852 and 3600 °C, respectively, which means it cannot be destroyed under the usual carbonization conditions (= < 1000 °C) used for the synthesis of porous carbons. Additionally, MgO templating can induce the generation of a high surface area, large pore volume, and tunable pore size (mainly concentrated in mesoporous domains), and can be washed away with dilute acids such as hydrochloric acid (HCl) after the synthesis process.^[^ ^47^, ^48^ ^]^ Moreover, MgO templating can be combined with chemical activation, such as using KOH and other chemicals to generate greater hierarchical porosity composed of micro‐ and mesopores.^[^ ^49^ ^]^ Furthermore, it can also be combined with other template materials, such as ZIF‐8^[^ ^50^ ^]^ or Pluronic F127,^[^ ^51^ ^]^ to devise dual templating strategies. Porous carbons can be templated with MgO using different experimental or precursor variants, which are described and compared below to provide critical insights.

#### 2.1.1 “Ex Situ” MgO Templating

**Figure 3:**
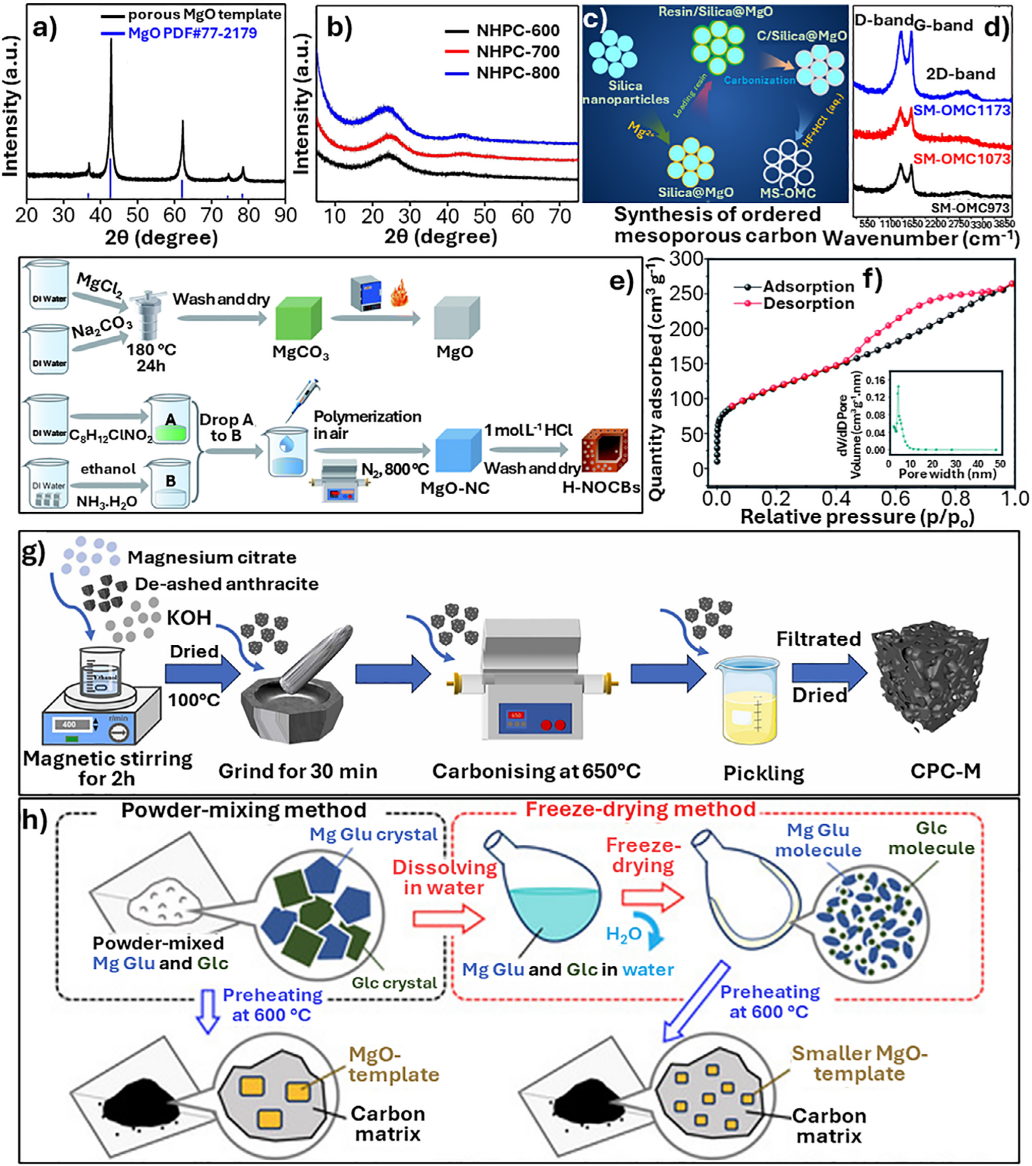
a,b) XRD of ZnO template showing sharp peaks and XRD of porous carbons showing absence of sharp peaks, indicating successful removal of ZnO after acid wash, Reproduced with permission^[^ ^32^ ^]^ Copyright Elsevier 2022, c,d) synthesis schematic and Raman spectra of ordered mesoporous carbon synthesized from a SiO_2_@MgO templated phenolic resin, Reproduced with permission^[^ ^52^ ^]^ Copyright Elsevier 2022, e,f) synthesis schematic of hollow N/O dual‐doped carbon boxes from polydopamine using MgO templating and its N_2_ sorption isotherms and pore size distribution curves, Reproduced with permission^[^ ^53^ ^]^ Copyright Royal Society of Chemistry 2022, g) synthesis schematic of coal‐based porous carbon using magnesium citrate‐based “in situ” MgO templating, Reproduced with permission^[^ ^61^ ^]^ Copyright Elsevier 2025, and h) synthesis schematic of hard carbon form sucrose and magnesium gluconate via MgO templating method, Reproduced with permission^[^ ^62^ ^]^ Copyright Wiley 2021.

Anionic polyacrylamide interacts electrostatically with one‐quarter quantity of the MgO template during high‐temperature carbonization to produce porous carbons (NHPCs) with decent surface areas of ≈547, ≈720, and ≈989 m^2^ g^−1^ at 600, 700, and 800 °C.^[^ ^32^ ^]^ A hierarchical porous structure dominated by micropores as well as meso and macropores was observed. According to XRD data, the NHPCs contained no residual MgO (**Figure** **3**a,b), indicating that the acid wash completely removed it. The NHPC‐700 material performed exceptionally well as a supercapacitor electrode due to its porous structure, high surface area, and heteroatom doping. However, in this synthesis, the MgO template was fabricated in a prior step involving sonication, 20 h of refluxing, and calcination at 500 °C. It was then used as a template with anionic polyacrylamide. This can be a significant time‐and cost‐consuming factor. Through an “ex‐situ” process, MgO can be used in conjunction with SiO_2_ to impart mesoporosity and order in porous carbons. Guo et al. utilized SiO_2_ with a diameter of 35 nm and coated it with MgO by calcination at 500 °C for 4 hr.^[^ ^52^ ^]^ The coated SiO_2_@MgO was first thermopolymerized at 100 °C with a phenolic resin to generate structural rigidity. It was then carbonized at 700, 800, and 900 °C, and finally washed with HCl and HF to obtain ordered mesoporous carbon (Figure 3c). Interestingly, MgO not only exerted a templating function but also induced catalytic graphitization, as evidenced by the development of a 2D band in addition to the typical D and G bands in the Raman spectra as the carbonization temperature increased (Figure 3d). In terms of porosity, the materials prepared at 800 °C exhibited a larger surface area and pore volume (1115 m^2^ g^−1^/1.83 cm^3^ g^−1^) than those prepared at 700 °C (896 m^2^ g^−1^/2.55 cm^3^ g^−1^) or 900 °C (879 m^2^ g^−1^/1.79 cm^3^ g^−1^). The influence of carbonization temperature is always crucial in the synthesis of porous carbon‐based materials, as an optimum temperature is required to maintain appreciable porosity and adequate surface functionalization with heteroatoms, which are crucial from an application point of view. The above two examples show that MgO can serve both as a templating agent to generate porosity and as a catalyst to create graphitic domains in the carbon structure.

An interesting study was reported by Zheng et al., who used a MgO template to synthesize hollow N/O dual‐doped carbon boxes as anodic materials for potassium ion batteries (KIBs).^[^ ^53^ ^]^ First, the MgO template with a box‐shaped morphology was synthesized using an aqueous mixture of magnesium chloride and Na_2_CO_3_ by hydrothermal treatment at 180 °C, followed by calcination at 600 °C. The application of hydrothermal carbonization (HTC) for 24 h may have enabled controlled nucleation and growth of magnesium carbonate (MgCO_3_), which, upon calcination, yielded MgO with a cubic, box‐shaped morphology, consistent with MgO's natural tendency to form a face‐centered cubic structure. Polydopamine was then thermopolymerized onto a MgO template, and the combination was carbonized at 800 °C, followed by acid washing to obtain hollow N/O‐doped carbon boxes (H‐NOCBs) (Figure 3e). The effect of MgO on porosity was evident from the N_2_ sorption isotherms, which showed that the H‐NOCBs had good surface area (≈410 m^2^ g^−1^) and mesopores with an average size of 3.93 nm (Figure 3f). The hollow box morphology, porosity, and N/O doping contributed to improvements in conductivity, active sites, durability, and volume expansion damping, making the H‐NOCBs function promisingly as anodes for potassium‐ion batteries (43 mAh g^−1^/5000 mA g^−1^).

A recent remarkable study by Bu et al. (2022) has shown that the MgO template can be recycled via a green route.^[^ ^54^ ^]^ They used basic magnesium carbonate (4MgCO_3_.Mg(OH)_2_.4H_2_O) as a template, which reacted with Mg powder at 800 °C to produce a composite of carbon and MgO (C@MgO). The MgO was then separated from the templated carbon (TC) by a reaction at room temperature with CO_2_ gas, resulting in the formation of Mg(HCO_3_)_2_, which was converted back to 4MgCO_3_·Mg(OH)_2_·4H_2_O by heating to 80 °C. This recycling of MgO is a great advantage compared to its loss by the usual acid etching process. In addition, the process proved to be highly feasible in terms of reproducibility over 10 cycles and the TC performed exceptionally well as a supercapacitor electrode material in aqueous (KOH/H_2_O, >154 F g^−1^) or organic (Et_4_NBF_4_/AN, >140 F g^−1^) and ionic liquids (EMIMBF_4_, >178 F g^−1^) electrolytes. Overall, the two main advantages in terms of cost reduction due to the repeated use of the MgO template and zero emissions, as no harmful by‐products are released during the templating process, are desirable, together with the scientific appeal of the reliable properties and application performance of the materials. Recently (2024), Zheng et al., although not focusing on MgO recycling, utilized 4MgCO_3_.Mg(OH_2_).5H_2_O as a MgO template, and fabricated HPC for supercapacitors.^[^ ^55^ ^]^ They introduced the novelty of co‐carbonizing a previously ball‐milled mixture of 4MgCO_3_·Mg(OH_2_)·5H_2_O and potassium citrate tribasic monohydrate (K_3_C_6_H_5_O_7_·H_2_O – source of carbon and chemical activation) at 800 °C. The MgO template derived from 4MgCO_3_.Mg(OH_2_).5H_2_O serves as a scaffold for the deposition and growth of carbon from K3C6H5O7·H2O during high‐temperature carbonization. With the help of the MgO support, carbon can form chains and graphene fragments and be chemically activated by volatiles released during carbonization. Consequently, the templating effect of MgO led to an optimized hierarchical porous carbon consisting of micro, meso, and macropores with a reasonable surface area of 1769 m^2^ g^−1^. The material delivered an impressive electrical double layer capacitance (EDLC) based specific capacitance of 305.5 F g^−1^ at 0.5 A g^−1^ in a three‐electrode system.

Jiao et al. (2025) found that the incorporation of nano‐MgO template in honey produces porous carbon nanosheets that possess porosity lying predominantly in the meso and macroporous range, and they further analyzed the impact of porosity on supercapacitive behaviour.^[^ ^56^ ^]^ Five materials (HPC‐1 to 5) were prepared, with the honey weight held constant and the MgO weight varying from 1 to 5 g. With increasing MgO weight, the porous carbons showed increases in mesopore area and volume, with HPC‐1 showing ≈10% mesopore area and HPC‐5 showing ≈70% mesopore area. The mesopore volume also increased from ≈35% to 95%. This directly impacted the performance of materials in a three‐electrode supercapacitor system with HPC‐5 delivering 359 F g‐1 at 0.5 A g^−1^, which was much higher than HPC‐1 with 110 F g^−1^ at a similar current density. The rate capability of the materials also varied in this fashion, highlighting the significance of mesoporosity on electrochemical performance. HPC‐5 also retained 63% capacitance even at a high current density of 50 A g^−1^. Furthermore, the charge‐transfer resistance from EIS decreased gradually from HPC‐1 to HPC‐5, further highlighting the significance of mesopores in the structure. The authors claimed that the higher mesoporosity accounts for more open channels for faster ion/electron transfer. And hence HOC‐5 showed the best performance. This study demonstrates that a ≈60% increase in mesopore area and volume resulted in a 226% increase in capacitance.

#### 2.1.1 “In Situ” MgO Templating

Commercial nano‐powder of ZnO has frequently been used for “in situ” templating.^[^ ^51^ ^]^ However, it could be an expensive venture for the templating process. Alternatively, using a magnesium salt as a MgO template source during high‐temperature carbonization with a carbon resource is a suitable approach to avoid the time‐consuming multistep synthesis of MgO. The MgO produced “in situ” is more likely to be consumed during the carbonization process itself, and any residues of it could be removed by conventional acid etching with dilute acids, the most common acid being HCl. Lighter magnesium slats, such as magnesium hydroxide Mg(OH)_2_^[^ ^57^ ^]^ and MgCl_2,_^[^ ^58^ ^]^ and MgCO_3_^[^ ^59^ ^]^ etc., are well‐suited for the “in situ” formation of MgO template during the synthesis of porous carbons. Heavier salts, such as organic magnesium salts, serve as suitable precursors for the development of porous carbon materials mediated by an in situ MgO‐based templating effect. Magnesium citrate, acetate,^[^ ^60^ ^]^ ascorbate, glycinate, lactate, and malate, etc., fall into this category. The MgO templating effect leads to the generation of porous structures and, to a certain extent, graphitization in the carbon matrix. Feng et al. applied the “in situ” MgO templating strategy to synthesize porous carbons from coal and used them as anodic materials for microbial fuel cells.^[^ ^61^ ^]^ The de‐ashed anthracite obtained from coal by acid etching was mixed in water with magnesium citrate in different proportions (0, 0.5, and 2 g), carbonized at 650 °C, and acid‐washed to obtain catalytic coal‐based porous carbon materials (CPC‐M0.5‐2) (Figure 3g). In terms of porosity, reasonable surface areas were observed (CPC‐M – ≈859, CPC‐M0.5 – ≈1056, and CPC‐M2.0 – ≈1150 m^2^ g^−1^), and a trend toward a higher mesoporous fraction with increasing salt loading was observed. MgO templating also resulted in morphological changes: CPC had fewer pores, CPC‐M0.5 had a smoother surface with numerous pore structures, and CPC‐M2.0 had a rougher surface. CPC‐M0.5 was the optimal material and showed signs of catalytic graphitization by the “in situ” generated MgO. Although “in situ” MgO templating offers significant advantages in terms of simplicity and time efficiency, limited control over the structural rigidity of the MgO template can lead to the formation of less‐ordered amorphous materials.

Bulky magnesium precursors, such as magnesium gluconate, can also be used to source MgO templating nanostructures, which can then be used to develop hard carbons with appreciable porosity.^[^ ^62^ ^]^ The synthesis process for the optimized hard carbon involved freeze drying a mixture of known amounts of magnesium gluconate and sucrose (carbon source), preheating at 600 °C to form nano MgO in the carbon matrix, and finally carbonization at 1500 °C, followed by acid washing to obtain MgO‐templated hard carbon (Figure 3h). For comparison, the materials were also synthesized as powders by physical mixing, yielding larger MgO template sizes than with freeze‐drying. Molecular dynamics calculations using first‐principles methods show that the pore structure of the hard carbon remained stable after removal of the MgO template by acid washing. From an application perspective, these hard carbons proved to be beneficial electrode materials for sodium‐ion batteries (SIBs). They provided a reversible capacity of 478 mAh g^−1^ with a coulombic efficiency of 88%.

**Figure 4:**
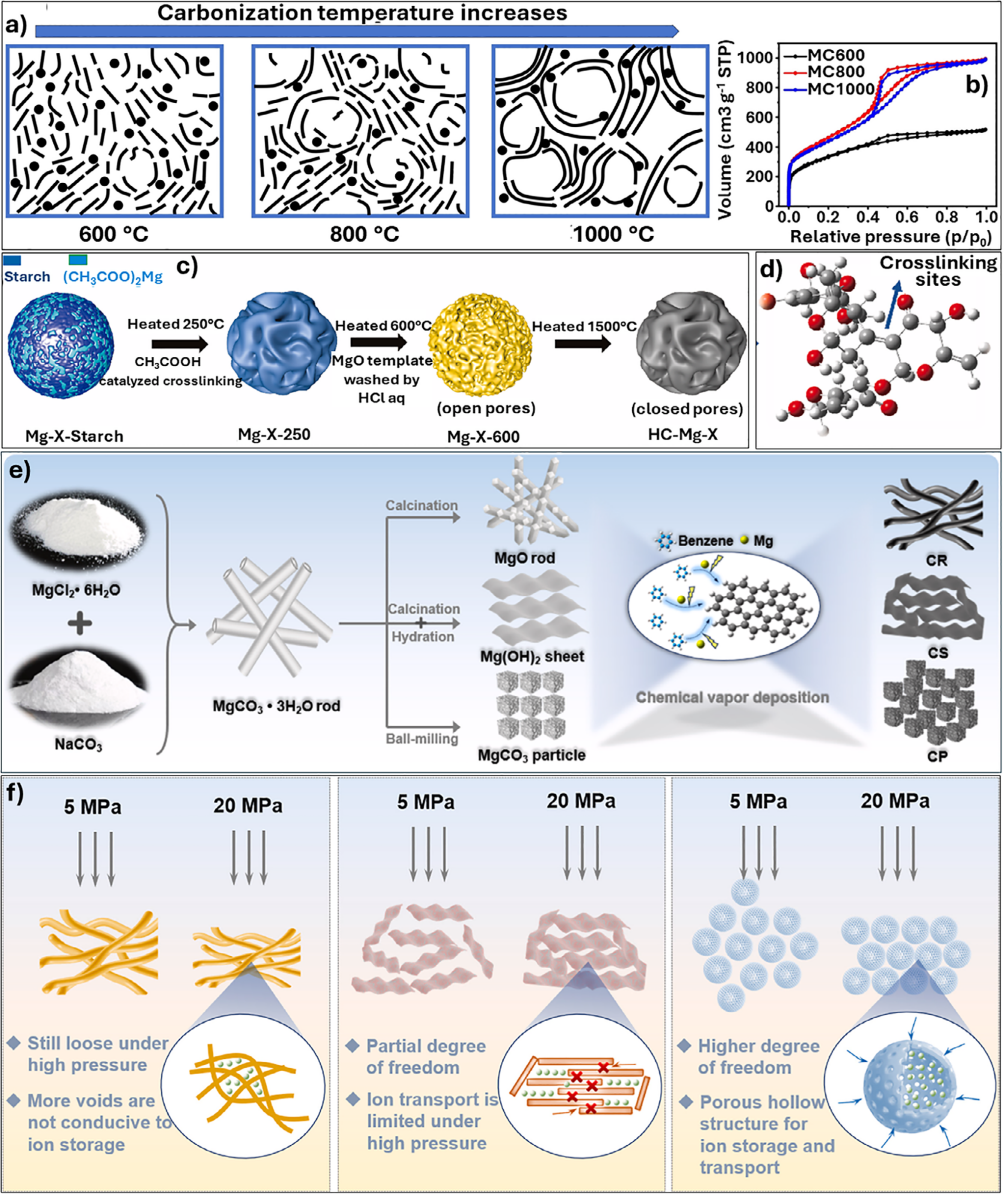
a,b) Graphitic domains and pore structure evolution of MgO templated porous carbons obtained from direct carbonization of magnesium citrate, and their N_2_ sorption isotherms indicating the porosity development across micro and mesoporous domains, Reproduced with permission^[^ ^63^ ^]^ Copyright Wiley 2025, c,d) synthesis schematic involving the dual functionalization of cross‐linking of starch and MgO templating using magnesium acetate, and cross‐linking sites for starch molecules, Reproduced with permission^[^ ^64^ ^]^ Copyright Elsevier 2025, and e,f) synthesis schematic of three different types of rods (CR), sheets (CS), or particles (CP) morphologies of carbon derived from benzene via MgO templating, and others, and the impact of morphology on the ion storage in a hybrid capacitor, Reproduced with permission^[^ ^65^ ^]^ Copyright Elsevier 2025.

Recently (2024), Ma et al. carbonized magnesium citrate as a stand‐alone precursor and obtained graphitized mesoporous carbon, which was employed as an electrode material for lithium‐ion capacitors (LICs).^[^ ^63^ ^]^ At various carbonization temperatures of 600, 800, and 1000 °C, an ordered pore structure with considerable N_2_ sorption and surface area (MC600 – 1187 m^2^ g^−1^, MC800 – 1673 m^2^ g^−1^, MC1000 – 1569 m^2^ g^−1^) was observed within micro and mesoporous domains, which was possible due to the templating effect of the “in situ” generated MgO (**Figure** **4**a,b). The impact of carbonization temperature on the carbon structure was evident, with a higher threshold of 1000 °C facilitating the development of long‐range graphitic domains that conferred higher conductivity to this material (MC1000 – 874.9 S m^−1^) than those synthesized at 600 or 800 °C (MC600 – 8.9×10^−5^ S m^−1^ and MC800 – 118.1 S m^−1^). In terms of application, MC800 provided a reasonable reversible lithium capacity of 110.51 mAh g^−1^/0.25 A g^−1^. As a LIC, impressive energy (152.2 Wh kg^−1^) and power densities (14.3 kW kg^−1^), were achieved with 80 % capacity retention after 5000 cycles at 1 A g^−1^. An interesting aspect of MgO templating based on the organic salt magnesium acetate was recently described by Huang et al. (2025) in the context of improving the initial coulombic efficiency (ICE) of SIBs by converting open to closed pores.^[^ ^64^ ^]^ They observed that the magnesium acetate initially releases acetic acid at 250 °C, which cross‐links the starch molecules. When the cross‐linked structure is heated to 600 °C, the template‐forming effect of ZnO creates an open‐pored carbon network. Later, the open pores can be closed at their ends by carbonization at 1500 °C (Figure 4c,d). It has been shown that the closed‐pore architecture improves the electrochemical performance of the materials, particularly the ICE. The ICE was increased from 74.78% in an open‐pore framework to 84.68% in a closed‐pore framework. The open and closed pores were regulated by using an optimal amount of magnesium acetate; lower amounts led to structural instability, while higher amounts led to the formation of open pores. Open pores allow easier electrolyte penetration, and exposure to a larger surface area triggers the formation of an excessive SEI layer, resulting in a lower ICE. Closed pore, on the other hand, limits access to the surface and can lead to the formation of a more stable, thinner SEI layer, thereby minimizing sodium ion loss. The material with closed pores also exhibited a higher reversible capacity of 369.17 mAh g^−1^, compared to 264.98 mAh g^−1^ for the material with an open pore structure. Again, the closed pores could help reduce irreversible sodium‐ion entrapment, thereby increasing the reversible capacity. Morphological control is also an essential feature of MgO templates, influencing the performance of the final porous carbons for electrochemical applications. MgCO_3_.2H_2_O rods synthesized by a liquid‐phase growth method using MgCl_2_.6H_2_O and sodium carbonate (Na_2_CO_3_) were converted into various morphologies, including MgO rods, Mg(OH)_2_ sheets, and MgCO_3_ particles, by calcination, calcination with hydration, and ball milling, respectively.^[^ ^65^ ^]^ Subsequent high‐temperature carbonization of the three morphologically distinct materials and their acid pickling replicated their morphologies to the final carbon materials, which were obtained as either rods (CR), sheets (CS), or particles (CP) (Figure 4e). In terms of porosity, the MgO rods exhibited meso‐ and macroporous features, while the sheets and particles showed a structure enriched with micro, meso, and macropores. Pressure‐based electrochemical tests showed that the CP‐based electrode exhibited a 14% increase in gravimetric specific capacitance when the pressure was increased from 5 to 20 MPa. This contrasts with CR or CS, where the specific capacitance decreases with increasing pressure (Figure 4f). In all three cases, the base template formed during high‐temperature carbonization is MgO. The overall investigations highlight how different morphologies can create different pore domains of micro, meso, and macropores, allowing for the handling of pressure in a different way during electrochemical testing.

### 2.2 Iron Oxides (Fe2O3 and Fe3O4)

Iron‐based oxides, including Fe_2_O_3_ and Fe_3_O_4_, are another class of promising hard inorganic sacrificial templates for the synthesis of porous carbons.^[^ ^66^ ^]^ These oxides can be uniformly dispersed in the carbon precursors during high‐temperature synthesis and later removed by mild acid etching. Their templating effect can create hierarchical porous structures spanning micro, meso, and macro domains in the synthesized porous carbons, which is critical from an application perspective.^[^ ^67^, ^68^, ^69^ ^]^ In addition to creating porosity, these oxides, like MgO, are also capable of introducing graphitic domains into the carbon matrix. This is an additional property that is highly desired in electrochemical usage, including supercapacitors, batteries, and electrocatalysis.^[^ ^70^ ^]^ Overall, these oxides provide a sustainable and scalable method for developing porous carbon with the desired porous properties suitable for specific applications. This section compiles recent research papers on the role of iron‐based oxides as templates for porous carbons, with discussions that provide critical analysis and valuable insights.

**Figure 5:**
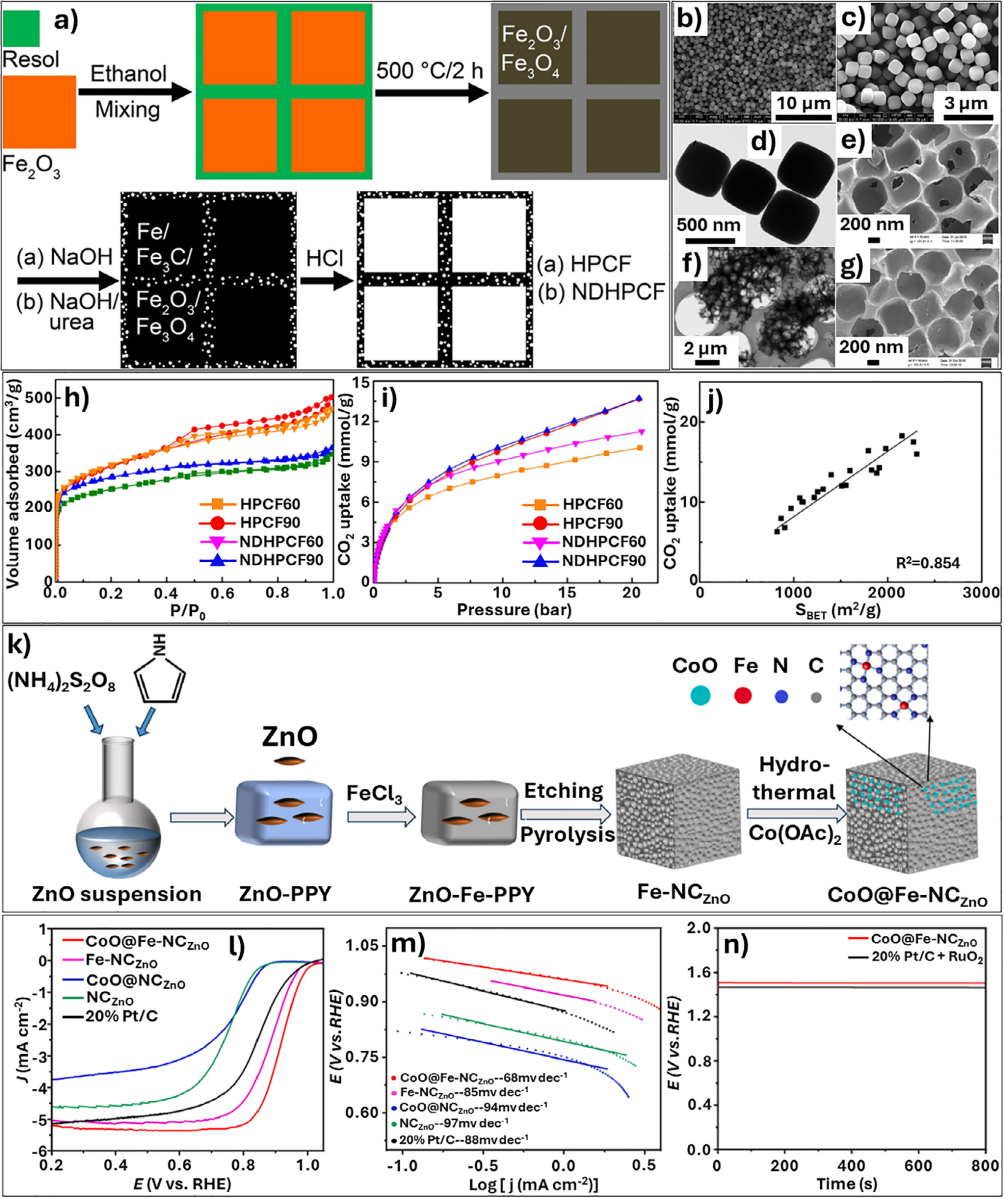
a) Schematic for the synthesis of HPCF and NDHPCF materials, b–d) SEM and TEM images of Fe_2_O_3_ template, e,f) SEM and TEM images of HAPCF60, h) N_2_ sorption, i) CO_2_ uptake at 0 °C, and j) linear fitting of CO_2_ adsorption with surface area, Reproduced with permission^[^ ^28^ ^]^ Copyright Elsevier 2020, k) Schematic for the synthesis of CoO@Fe‐NC_ZnO_ catalyst and the mechanism of pore formation, l) LSV curves, m) Tafel plots, and n) open circuit voltage curves for various catalysts, Reproduced with permission^[^ ^71^ ^]^ Copyright Elsevier 2025.

A hierarchical porous carbon framework (HPCF) was developed using a combined strategy of Fe_2_O_3_‐based nano‐templating and sodium hydroxide (NaOH)‐based chemical activation.^[^ ^28^ ^]^ The cubic macroporous structure of Fe_2_O_3_ pre‐synthesized by HTC was successfully replicated onto a high surface area porous carbon. To synthesize porous carbon, a resol was treated with pre‐synthesized cubic Fe_2_O_3_ at 50 °C to form a gel, which was then heated at 100 °C to create a hard red product. This product was then carbonized at 500 °C under inert conditions. The resulting carbonized product was then chemically activated with NaOH to obtain the activated material (**Figure** **5**a). Instead of removing inorganic impurities with dilute HCl at room temperature, a hydrothermal treatment with 37 wt% HCl at 120 °C for 3 h was performed to obtain HPCF. A nitrogen‐doped version of HPCF (NDHPCF) was also prepared by using urea as a precursor alongside NaOH during chemical activation. Scanning electron microscopy and transmission electron microscopy (SEM and TEM) images confirmed the cubic shape and nanometre size (> 500 nm) of the Fe_2_O_3_ cubes prepared with HTC (Figure 5b‐d). After activation with NaOH, the cubic morphology can be maintained in the porous carbon structure of the HPCFs with macropores of 680–750 nm (Figure 5e–g). XRD and Raman spectra proved the amorphous structure of the materials. The surface area of the templated porous carbons ranges from 866 to 1100 m^2^ g^−1^. Specifically, materials synthesized with a 90 min activation time (HPCF90) exhibit a larger surface area than those synthesized with a 60 min activation time (HPCF60). The N_2_ adsorption isotherms show a mixture of micropores and mesopores and are of types I and IV (Figure 5h). The pore size was determined using density functional theory (DFT), and two sharp peaks at 0.5 and 0.7 nm were identified as the dominant features. The materials were tested for CO_2_ adsorption, and NDHPCF60 was found to adsorb a maximum of 2.76 mmol g^−1^ at 1 bar and 25 °C, a value considered reasonable in the published literature. Both micropores and nitrogen doping influenced CO_2_ adsorption. The CO_2_ adsorption of the materials under high pressure at 0 °C is also reasonable (Figure 5i). The heat of adsorption values indicate physical adsorption (23.4 kJ mol^−1^); the N_2_ sorption was low compared to CO_2_, and the CO_2_: N_2_ (15:85) selectivity of the material was within 16–20. A linear correlation was found between the CO_2_ adsorption of the studied materials and the surface area (Figure 5j). Although the outcome of this study, in terms of improved structural integrity of the synthesized materials, is promising, the multiple synthesis steps, including separate Fe_2_O_3_ pre‐synthesis, could be an obstacle to the materials' scalability.

Song et al. have recently (2025) explored a novel secondary templating strategy using Fe and Fe_3_O_4_ nanoparticles to impart rigidity to the porous carbon‐based catalysts (CoO@Fe‐NCZnO) composed of single atomic FeNx and CoO nanoparticles.^[^ ^71^ ^]^ The synthesis involved a multistep reaction in which ZnO was first used as a primary template to form a ZnO‐PPY‐based material, which was pyrolyzed with FeCl_3_. Later, the product was treated with Co(OAc)_2_ to produce CoO@Fe‐NCZnO (Figure 5k). The authors reported that the pyrolysis of FeCl_3_ leads to the formation of Fe and Fe3O4 nanoparticles, which imparted stability to the porous structure of the catalyst and their removal by acid etching can lead to the generation of a higher surface area of 1143 m^2^ g^−1^, which was much larger than that of the unetched material (11 m^2^ g^−1^) or the washed product carbonized without FeCl_3_. It is a common practice to wash carbonized materials with acid to remove residual material from the pores. The stable porous structure and good conductivity of the catalyst, resulting from the primary templating effect of ZnO and the secondary templating effect of Fe and Fe_3_O_4_ nanoparticles, resulted in good performance in ORR and oxygen evolution reaction (OER), where a half‐wave potential (E1/2) of 0.917 V and an onset potential of 1.001 V were observed, which was higher than the commercial Pt/C (0.863 V and 0.975 V) (Figure 5l). The Tafel slope for the catalyst (68 mV dec^−1^) was also the lowest among the materials investigated, including the commercial Pt‐based catalyst (Figure 5m). In addition, the catalyst proved to be an efficient electroactive material for zinc‐air batteries, providing an open‐circuit voltage of 1.52 V, which is higher than that of 20% Pt/C + RuO_2_ (Figure 5n).

While it is possible to fabricate porous carbon templates using Fe or its oxides in the Fe_2_O_3_ and Fe_3_O_4_ forms, it is also common to use porous carbon as an anchor to hold such nanoparticles on its surface, which again is critical from an application perspective.^[^ ^72^ ^]^ However, this is possible either via “ex‐situ” modification of the porous carbon with minute amounts of a suitable iron precursor at a high carbonization temperature, which does not require acid etching, or by washing the final carbonized product with alternatives or storing it as such, which could compromise porosity. Peng et al. proposed a unique one‐step carbonization strategy, in which SiO_2_ at 800 °C provides a templating effect, NaNO_3_ provides an activation effect, and iron nitrate in non‐hydrated form generates Fe_2_O_3_ nanoparticles on the surface of porous carbon.^[^ ^73^ ^]^ The silica etching with 2M NaOH does not affect the Fe_2_O_3_ nanoparticles generated on the surface, and the catalyst was found to be beneficial for activating peroxymonosulfate (PMS), which can degrade organic pollutants. As the focus of this review article is on metal oxides, such as iron, as templates, the surface decoration of iron oxides on porous carbons is not discussed in detail.

**Figure 6:**
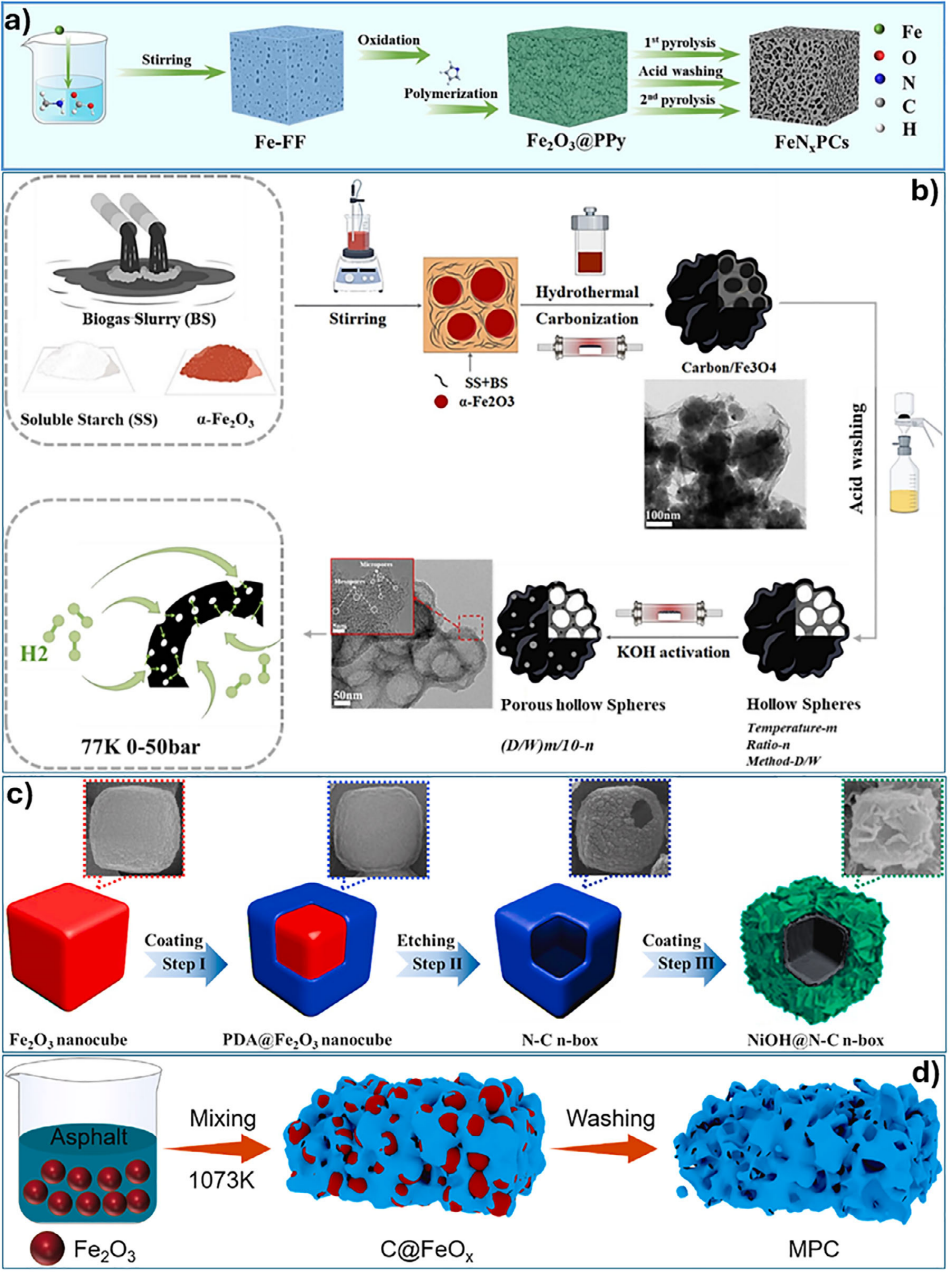
Synthesis schematics of various types of Fe_2_O_3_ templated porous carbon materials a–d) Fe–N_x_ doped porous carbon‐based ORR catalyst, Reproduced with permission^[^ ^74^ ^]^ Copyright Elsevier 2023, biogas slurry, and starch‐derived hierarchical hollow carbon spheres, Reproduced with permission^[^ ^75^ ^]^ Copyright Elsevier 2025, hierarchical Ni(OH)_2_ nanosheets grown on hollow nitrogen‐doped carbon nanoboxes, Reproduced with permission^[^ ^76^ ^]^ Copyright Elsevier 2022, and mesoporous carbons derived from spent asphalt, Reproduced with permission^[^ ^77^ ^]^ Copyright Elsevier 2021.

In addition to providing the 3D templating effect to create porous structures with the carbon matrix, Fe_2_O_3_ can also provide Fe^3+^ ions to facilitate polymerization of carbon precursors such as pyrrole.^[^ ^74^ ^]^ Fe_2_O_3_ template synthesized from the oxidation of an iron formate framework (Fe‐FF) built using the FeCl_2_.4H_2_O and polyvinylpyrrolidone was copolymerized with pyrrole (Fe_2_O_3_@PPy) at 800 °C to form Fe–N_x_ doped porous carbon catalysts (FeN_x_PCs) with 3D crosslinked pore structure (**Figure** **6**a). The cubic Fe_2_O_3_ contributed to templating: initially, pyrrole was adsorbed onto its surface via ultrasonication, and its subsequent decomposition produced Fe^3+^, which drove the polymerization of pyrrole. Although the finally prepared catalyst FeN_x_PCs contained iron in its structure, there was no evidence that iron played any role in graphitizing the carbon, which was amorphous as determined by microscopic examination. The catalyst turned out to be a good material for ORR in zinc‐air batteries. In another instance, an industrial α‐Fe_2_O_3_ template induced the development of a hollow carbon structure derived from a mixture of biogas slurry and starch via HTC, which was then transformed into hierarchical porous carbon (containing micro, meso, and macropores) with high surface area using the KOH‐based chemical activation (Figure 6b).^[^ ^75^ ^]^ Owing to their high surface area of 3134 m^2^ g^−1^, large pore volume of 2.0 cm^3^ g^−1,^ and 88% microporosity residing within the pore size range of 0.74‐1.18 nm, the materials demonstrated exceptional H_2_ storage capacity of 6.73 wt% at 77K and 50 bar. The nanocubic structure of porous carbons developed using an Fe2O3 template is also an exciting option for building or wrapping various nanosheet‐type materials around cubic shapes to devise a material of interest for biosensing.^[^ ^76^ ^]^ The Fe_2_O_3_ template formed by the HTC‐based reaction of FeCl3 and NaOH was used to surface‐coat polydopamine (PDA), and its carbonization at 500 °C produced N‐doped carbon nanoboxes (Figure 6c). These hollow nanoboxes served as ideal substrates for coating a thin Ni(OH)_2_ layer on their surfaces, forming hollow Ni(OH)_2_@N‐C‐n boxes that acted as effective electrochemical sensors for alpha‐fetoprotein cancer biomarkers. Commercially available nano Fe_2_O_3_ (particle size of 30 nm) can also be used to create an interconnected 3D porous structure, high surface area, and numerous crystal defects in porous carbon derived from spent asphalt (Figure 6d).^[^ ^77^ ^]^ In most of the above templating pathways, Fe_2_O_3_ or its remnants can be removed from the pores formed during high‐temperature carbonization using a dilute acid wash. More importantly, the acid wash can be tailored to retain Fe_2_O_3_ or Fe nanoparticles within the carbon matrix, serving as catalytic sites for various reactions depending on application requirements. Overall, Fe_2_O_3_ as a template offers enormous potential for the design of hollow porous carbons with interconnected pore structures, the quality of which can be further modified through chemical activation.

### 2.3 MnO2

Manganese oxide as a sacrificial hard template offers unique advantages attributed to its rich redox activity, morphological diversity,^[^ ^78^ ^]^ and, of course, the ease of removal with dilute acids. It can also serve as a reactive template during the polymerization of various precursors.^[^ ^79^ ^]^ In comparison to other surficial hard templates discussed, specific morphologies such as nanotubes, nanosheets, and nanorod structures can be replicated onto porous carbons via the utilization of MnO_2_ as a sacrificial hard template.^[^ ^80^ ^]^ It also allows precise control over the architecture of porous carbon, which can be tailored for specific applications in gas capture, energy storage, and electrocatalysis.

**Figure 7:**
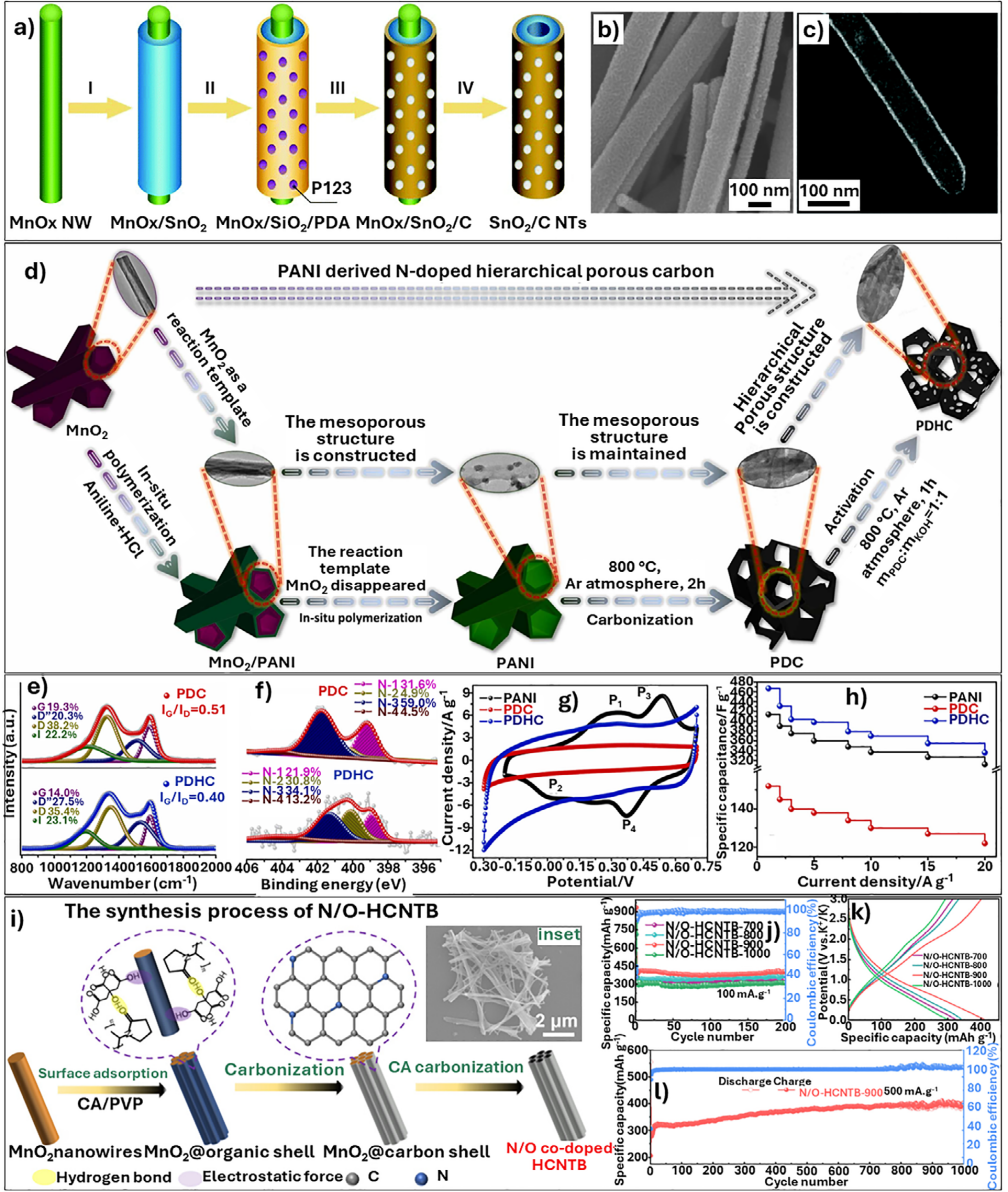
a) Schematic for the synthesis of SnO_2_/C nanotubes involving multi step procedure; i) MnO_x_ nanowires synthesized from KMnO_4_ via HTC was coated with SnO_2_ layer via further HTC treatment, ii) MnO_x_/SnO_2_ was encapsulated with polydopamine (PDA) to obtain MnO_2_/SnO_2_/PDA, iii) carbonization of MnO_2_/SnO_2_/PDA to form MnO_2_/SnO_2_/C, and iv) washing/etching with oxalic acid to remove MnOx and form SnO_2_/C, b) FESEM images of MnO_x_/SnO_2_/C nanotubes with varying diameter, c) STEM imaging to illustrate a 15 nm thick SnO_2_/C layer formation after the removal of MnO_x_ template, Reproduced with permission^[^ ^81^ ^]^ Copyright Royal Society of Chemistry 2016, d–h) MnO_2_ nanotubes templating (PDC) and KOH activation based synthesis schematic of PANI derived N‐doped hierarchical porous carbons (PDHC), Raman spectra, XPS spectra, CV curves and specific capacitance recorded at different current densities, Reproduced with permission^[^ ^27^ ^]^ Copyright Elsevier 2022, and i–l) synthesis schematic of N/O co‐doped 1D hard carbon nanotube bundles (N/O‐HCNTBs) via MnO_2_ nanosheets templating method, inset I is the SEM image of N/O‐HCNTB‐900, the cyclic performance, charge discharge profiles, and long‐term cycling performance of N/O‐HCNTBs for potassium ion storage, Reproduced with permission^[^ ^82^ ^]^ Copyright American Society of Chemistry 2025.

For instance, MnO_2_ nanowires, synthesized from HTC treatment (160 °C/9 h) of a solution mixture of potassium permanganate (KMnO_4_) and polyvinylpyrrolidone (PVP), were employed as a template to design tin oxide (SnO_2_)/C nanotubes (**Figure** **7**a) that delivered a reversible lithium‐ion storage capacity of 596 mAh g^−1^ after 200 cycles.^[^ ^81^ ^]^ Starting with the 40–190 nm wide nanowires of MnO_2_, a thin 15 nm layer of SnO_2_/C was retained after the elimination of MnO_2_ template nanowires by reaction with oxalic acid (Figure 7b and c).

Later, Zhou et al. (2022) demonstrated that MnO_2_ nanotubes can act as a self‐sacrificing template during the synthesis of hierarchical porous N‐doped carbon.^[^ ^27^ ^]^ The MnO_2_ nanotubes synthesized from KMnO_4_ via autoclaving at 150 °C were sacrificed during a chemical reaction with aniline under acidic conditions, which also induced a templating effect for the development of mesoporous polyaniline (PANI) nanotubes (Figure 7d). The carbonization of PANI nanotubes at 800 °C produced a non‐porous carbon (PANI‐derived carbon‐PDC), which was subsequently activated with KOH to generate a porous hierarchy spread across micropores and mesopores (PANI‐derived hierarchical carbon‐PDHC). While PDC, which demonstrated templating by MnO2, had a lower surface area of 176 m^2^ g^−1^, it increased to 2040 m^2^ g^−1^ after KOH activation. However, PDC has fewer structural defects and greater graphitization, as evidenced by a lower I_G_/I_D_ ratio in the Raman spectra (Figure 7e). This was also confirmed by XPS, which indicated a decrease in nitrogen content in PDHC (6.8 at%) compared to PDC (2.3 at%), due to stronger activation conditions for PDHC (Figure 7f). When tested in a three‐electrode supercapacitor system, the cyclic voltammetry (CV) curves of PDC and PDHC showed near‐EDLC behaviour, contrasting with PANI, where more redox reactions occurred on the surface (Figure 7g). PDHC (467 F g^−1^), owing to its attractive features, surpassed PANI (414 F g^−1^) and PDC (152 F g^−1^) for specific capacitance at 1 A g^−1^ and other recorded current densities (Figure 7h). Very recently (2025), Wang et al. utilized MnO_2_ nanowires as a sacrificial template to produce N/O co‐doped 1D hard carbon nanotube bundles (N/O‐HCNTBs) for potassium storage.^[^ ^82^ ^]^ The MnO_2_ nanowires were synthesized in the first step via HTC treatment of an aqueous mixture of (NH_4_)_2_S_2_O_8_, MnSO_4_·H_2_O, and (NH_4_)_2_SO_4_ at 200 °C for 72 h, which was followed by its carbonization along with the carbon precursors of polyvinylpyrrolidone and citric acid (50:50 by wt) at 700–1000 °C and etching with citric acid and water to produce N/O‐HCNTBs (Figure 7i). The SEM imaging confirmed the nanotubes bundled together compactly (Figure 7i inset), and the material proved to be efficient for potassium storage by delivering a specific capacity of 407 mAh g^−1^ at 0.1 A g^−1^ (N/O‐HCNTB‐900) in the initial cycle and a good cycling capability with a specific capacity of 394 mAh g^−1^ at 0.5 A g^−1^ after 1000 cycles (Figure 7j‐l).

When considering MnO_2_, it is imperative to weigh the trade‐off between using it as a template and as a surface‐anchored or structurally incorporated domain within the porous carbon matrix. The latter use has been more prominent in the literature than the former. For instance, MnO_2_ nanospheres incorporated within an HPC can deliver a remarkable specific capacitance of 521 F g^−1^ at 0.5 A g^−1.[^ ^83^ ^]^ Such systems can benefit from MnO2's redox behavior in addition to EDLC contributions. From a practical point of view, using MnO_2_ as a template could be more expensive than some traditional templates, such as silicas or zeolites. However, very recently in 2025, Yu et al. also demonstrated that pre‐synthesized MnO_2_ nanosheets from manganese chloride (MnCl_2_) can be suitably utilized as a template to fabricate N‐doped hierarchical porous carbon nanosheets.^[^ ^23^ ^]^ These nanosheets proved to be conducive materials for both Na and K ion storage, reaching 433.9 and 523.7 mAh g^−1^ at 0.1 and 0.2A g^−1^, along with good cycling stability for 2000 and 3000 cycles, respectively. One instance reported that “in situ” generated MnO_2_ can be reduced by carbon into MnO, which then can act as a template to produce macropores and also assist in the generation of graphene‐like nanosheets in a porous carbon derived from anthracite, and the materials have been shown to deliver interesting results for zinc ion hybrid capacitors (ZIHCs).^[^ ^84^ ^]^

### 2.4 ZnO

ZnO is another low‐cost inorganic compound/material that is widely used as a template for creating porosity in carbon‐based materials.^[^ ^85^, ^86^ ^]^ Like other methods, any remnants left after the templating or porosity creation effect of ZnO will need to be eliminated from the pores via acid etching and repeated washings with water.^[^ ^87^ ^]^ Frequent use of dilute HCl for acid etching has been reported, with occasional instances citing other acids such as dilute acetic acid.^[^ ^88^ ^]^ There are also reports where ZnO can be eliminated via thermal decomposition and sublimation at high carbonization temperatures.^[^ ^89^ ^]^ The resultant porous carbon structure is dominated by meso and macropores.^[^ ^90^ ^]^ Micropores and mesopores may also be produced during the thermal evaporation of metallic zinc, which is formed from ZnO.^[^ ^91^, ^92^ ^]^ Moreover, hierarchy can be introduced into the porous texture of the carbons via an interplay between the quantities of ZnO used for templating and the amount of carbon precursor. However, the carbonization temperature for the mixture of the carbon precursor and ZnO exercises a dominant control over the generation of pores within micro, meso, or macro domains.^[^ ^93^ ^]^ ZnO does not lose its templating action even in the presence of chemical activating species for carbon, including the K‐based salts.^[^ ^94^ ^]^

**Figure 8:**
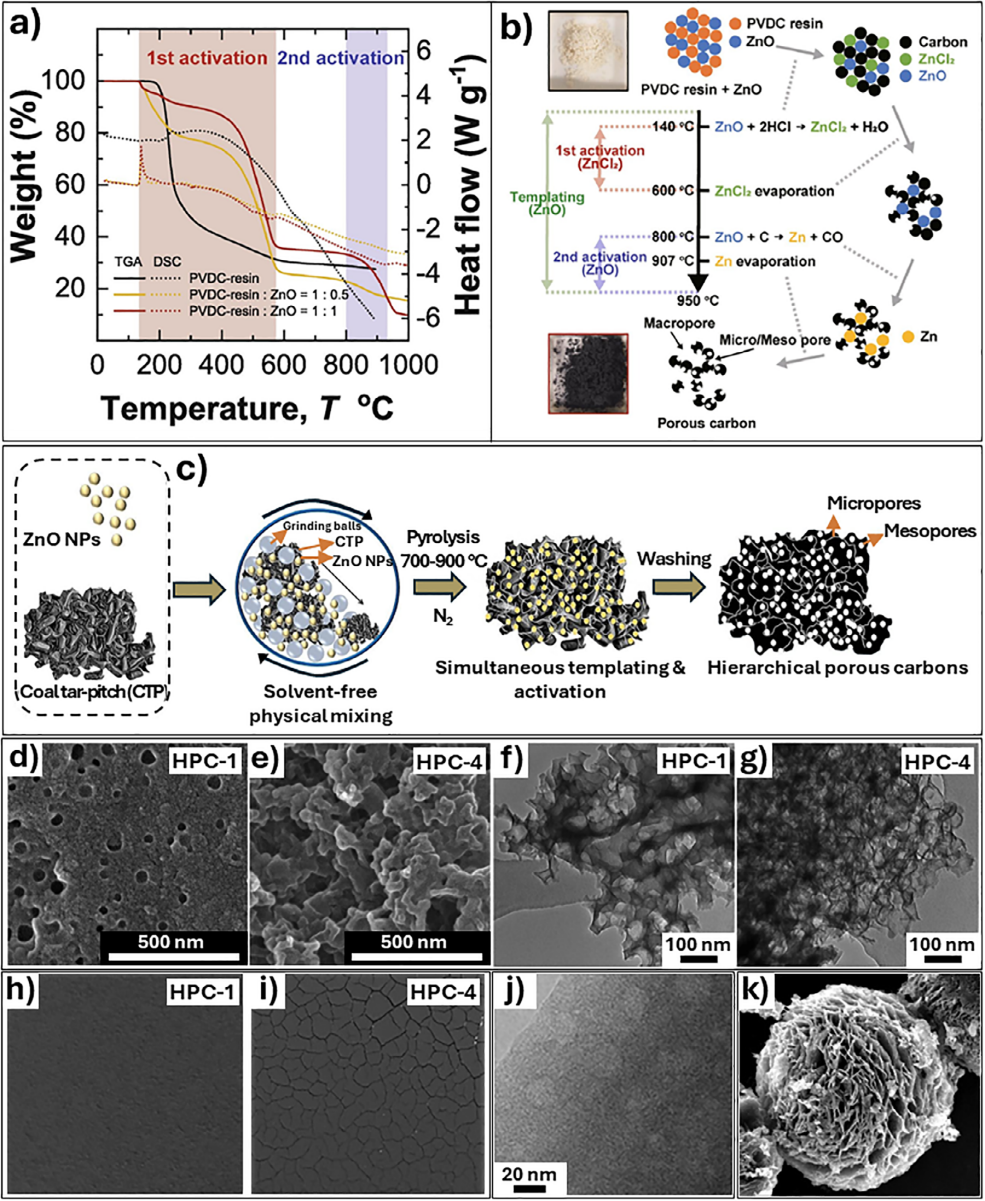
a) TGA profiles of various mixtures of ZnO and polyvinylidene chloride (PVDC) resin, and b) schematic of the mechanism of ZnO templating and chemical activation at various temperature regions, Reproduced with permission^[^ ^95^ ^]^ Copyright Elsevier 2021, c) synthesis schematic of hierarchical porous carbon from the templating action of nano ZnO on waste coal tar pitch, Reproduced with permission^[^ ^98^ ^]^ Copyright Elsevier 2024, d,e) SEM images, f,g) TEM images of HPC‐1 and HPC‐4 (hierarchical porous carbon synthesized from petroleum pitch, KOH and ZnO template, and h,i) their images after coating onto the stainless foil current collector highlighting the cracking and non‐cracking, Reproduced with permission^[^ ^99^ ^]^ Copyright American Chemical Society 2022, j) HR TEM Image of a porous carbon synthesized by utilizing the templating action of ZnO and activating action of KOH on water caltrop shell (pore size ≈20 nm is observed), Reproduced with permission^[^ ^100^ ^]^ Copyright American Chemical Society 2020, and k) Porous carbon sheet microspheres synthesized using ZnO template and KOH activation, Reproduced with permission^[^ ^101^ ^]^ Copyright Elsevier 2021.

Hwang et al. (2021) explored the mechanism of hard inorganic templating action of ZnO using a correlation between thermogravimetric measurements and energy‐dispersive X‐ray Spectroscopy (EDX).^[^ ^95^ ^]^ They reported that ZnO, at 140 °C, withdraws chlorine from the HCl produced from thermal decomposition of polyvinylidene chloride (PVDC) resin to produce ZnCl_2_ (ZnO + 2HCl → ZnCl_2_ + H_2_O), which subsequently produces micropore‐rich carbon through its thermal evaporation till 600 °C. Beyond 800 °C, any residual ZnO oxidizes the carbon (ZnO + C → Zn + CO) to yield more micropores, and the produced Zn evaporates at temperatures > 907 °C to create mesopores. The lower (< 600 °C) and higher (> 800 °C) temperature regions of the synthesis were termed as first and second chemical activation, respectively (**Figure** **8**a,b). Overall, the final material retained ZnO templated meso and macroporous sites while the porosity originated from the chemical action of ZnCl_2_ with an overall surface area of 2005 m^2^ g^−1^. The materials displayed reasonable specific capacitances of 201, 219, and 102 F g^−1^ in 6 M KOH, 1M sulfuric acid (H_2_SO_4_)_,_ and potassium sulfate (K_2_SO_4_) solutions, respectively. This study is also noteworthy for demonstrating that high‐temperature carbonization above the boiling point of metallic Zn (907 °C) is an effective way to obviate the need for acid or water washing, owing to the thermal evaporation of Zn metal during the process. Although it can also be concluded that the templating and activation effect of ZnO may differ if the carbon source is changed from a chlorinated compound to a non‐chlorinated one. For instance, PVP and Polyvinylidene fluoride (PVDF) based porous carbons synthesized using the commercial nano‐ZnO showed a smaller surface area of 873 m^2^ g^−1,^ even though the carbonization temperature was 1000 °C.^[^ ^96^ ^]^ The lack of ZnCl_2_ formation did not result in significant activation. However, the space‐confining or templating effect of ZnO resulted in a porous structure with the majority of mesopores centered at 40 nm. Moreover, this study highlighted that “in situ” generated ZnO via thermal decomposition of zinc acetate results in a greater number of micropores in the porous carbon structure. Overall, to gain an added advantage from ZnO templating and ZnCl_2_ activation, combining ZnO with a chlorinated compound is an ideal strategy for developing highly porous carbon materials.

The above studies corroborate the investigation of the mechanism of ZnO templating with earlier work by Xu et al. on two separate occasions.^[^ ^18^, ^97^ ^]^ Recently, the same group extended their work to apply the templating and activating effects of ZnO to a new precursor derived from waste coal tar pitch and reported porous carbons with a surface area of 1267 m^2^ g^−1^ and a generous mixture of micro‐ and mesopores.^[^ ^98^ ^]^ The synthesis began with ball milling ZnO nanoparticles and coal tar pitch in a defined ratio, followed by carbonization at 700, 800, and 900 °C. These carbonization temperatures were below the boiling point of metallic zinc (907 °C), necessitating a 3M HCl wash after carbonization to free the pores (Figure 8c). From an application point of view, a reasonable specific capacitance of 172 F g^−1^ at a current density of 0.1 A g^−1^ in 6 M KOH was observed.

The “in situ” generation of ZnO nanoparticles to create templating and activation effects is a prominent method reported in the literature. However, compared to commercial ZnO, which has a pore size in large mesopores (> 20 nm) and is primarily utilized in an “ex situ” pathway, the “in situ” generated ZnO generally produces micropores along with mesopores. A trade‐off exists due to the higher cost of the commercial ZnO as compared to the less expensive inorganic zinc salts. It is also important to note that, for commercial ZnO, the impregnation amount is a significant factor that directly controls the porosity of the carbon materials. In one instance, 900 °C carbonization of fixed amounts of petroleum pitch and KOH, along with a variable quantity of ZnO template, allows for a fine control over the distribution of micro, meso, and macropores within the porous carbon matrix.^[^ ^99^ ^]^ As per the SEM imaging observations, the lower amounts of ZnO (HPC‐1) lead to the generation of mesopores between the sizes of 20–60 nm, whereas the higher amounts (HPC‐4) lead to the complete breakdown of the carbon matrix into meso and macropores (Figure 8d,e). TEM imaging corroborated the SEM findings and further showed that mesopores and macropores are interconnected via micropores (Figures 8f,g). As a result of the extensive porosity produced in HPC‐4, the tap density was the lowest, which made this material unsuitable as an electrode material due to its potential cracking behaviour on the current collector as compared to HPC‐1, which was suitable for electrochemical operations due to its larger tap density (Figure 8h,i). Therefore, appropriate control over the amount of ZnO impregnation is significant from an application perspective as well. Similar observations regarding the multifaceted development of porosity were also reported by Hsu et al. in their work on water caltrop shells, which were simultaneously activated with KOH and templated with mesoporous ZnO.^[^ ^100^ ^]^ The commercially obtained ZnO, with a mesopore size of 20 nm, left a clear impression of its mesoporosity within the carbon matrix via space‐confinement effects, underscoring its role as a hard template (Figure 8j). Another report highlights that the unique microspherical morphology of basic zinc carbonate (Zn_5_(OH)_6_(CO_3_)_2_) can be replicated to an “in situ” formed ZnO, which is further transferred to the porous carbons derived from coal tar pitch (CTP).^[^ ^101^ ^]^ Zn_5_(OH)_6_(CO_3_)_2_ microspheres were synthesized initially via hydrothermal treatment of a mixture of urea and zinc nitrate hexahydrate at 100 °C. These microspheres were then coated onto CTP as ZnO microspheres via calcination. Lastly, the composite was activated with KOH at 700 °C to obtain porous carbon sheet microspheres (PCSMs). In addition to the replication of morphology onto PCMSs (Figure 8k), ZnO also served the purpose of creating a surface area of 1359.88 m^2^ g^−1^ and mesopores in the carbon structure with a size of 8.72 nm, and combining these features resulted in an impressive specific capacitance of 313 F g^−1^ at a current density of 1 A g^−1^ in 6M KOH electrolyte.

For the removal of ZnO template, either acid etching or thermal evaporation at temperatures > 907 °C are the currently reported methods. Acid etching is more energy‐efficient than thermal evaporation. At a laboratory scale, acid etching washing is less expensive as well. In the bigger picture, for the commercial production of ZnO‐templated carbons, acid etching could be a more beneficial option. Mineral acids, such as HCl, are relatively inexpensive in bulk compared to the energy required to operate the high‐temperature furnaces needed for large‐scale operations. Moreover, the spent acid could be neutralized, leaving a positive environmental footprint, and the dissolved zinc could be precipitated as Zn(OH)_2_ for reuse in similar or related operations. Overall, ZnO is a promising inorganic hard template for the synthesis of porous carbons that are used in various applications.^[^ ^102^ ^]^

**Figure 9:**
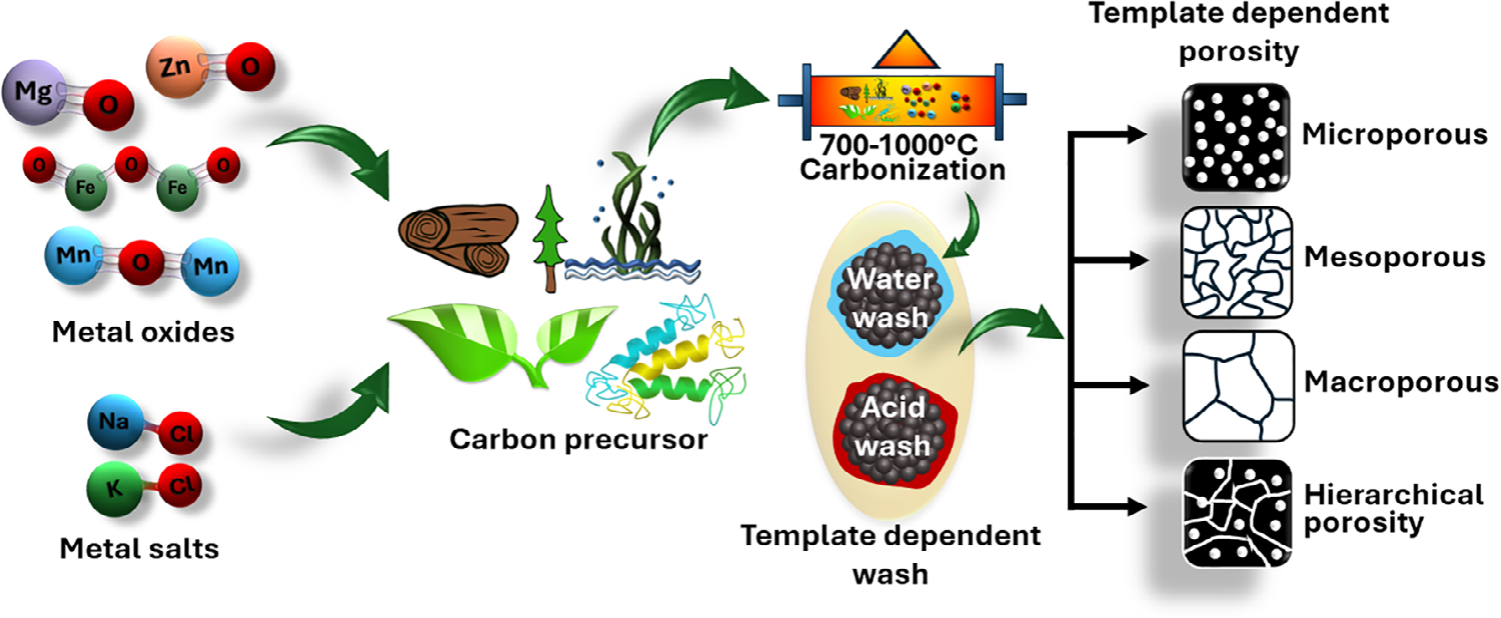
A general schematic of the preparation route of porous carbons via metal oxides and metal salts‐based templating.

Overall, the porosity induced by templating plays a crucial role in determining the application perspective of the porous carbons. A schematic of the process of synthesizing porous carbons with varied porosity from various carbon precursors and metal oxide/salt templates is shown in **Figure** **9**.

### 2.5 Comparative Insights Among Metal Oxide Templates

Metal oxides, including MgO, Fe_2_O_3_/Fe_3_O_4_, MnO_2_, and ZnO, share the advantages of high thermal stability, ease of removal via dilute acid etching, and the ability to induce porosity across multiple pore‐size domains. Their distinct physicochemical properties, however, can yield notable differences in the morphology, porosity, graphitic structure, and chemical functionalities of the resulting porous carbons, thereby directly influencing their application performance. A comparative evolution of these metal oxides is imperative to highlight their templating behaviour, porosity formation, and implications for targeted applications.

#### 2.5.1 Structural and Thermal Stability

MgO is highly stable up to temperatures well beyond 2000 °C, allowing it to retain rigidity and, in turn, replicate the pore structures in the resulting carbons. ZnO remains stable up to ≈900 °C; therefore, its volatilization at higher temperatures can lead to slight morphological distortions and the formation of interconnected micro‐meso frameworks. In contrast, Fe_2_O_3_ and MnO_2_, which are prone to partial phase transformation or reduction at high temperatures, can yield templating results that differ from expectations. For instance, Fe_2_O_3_ can be reduced to Fe_3_O_4_ or metallic Fe, which facilitates graphitization of carbon, and MnO_2_ can undergo reduction to MnO or decompose into lower oxides that alter the pore evolution into sizes larger than micropores.

#### 2.5.2 Templating Efficiency and Porosity Development

Metal oxides vary significantly in their ability to impart a porous architecture to porous carbons. MgO is known to produce meso‐macroporous carbons with a broader pore‐size distribution in the 3–50 nm range, which can be further controlled by particle size and impregnation amount. Such a pore size is conducive to higher mass transport, thereby facilitating the use of MgO‐templated carbon for applications such as supercapacitors and water purification. ZnO produces pores in the 1–5 nm range, and such a fine balance between micro and mesopores is ideal for applications such as gas storage and electrocatalysis. Iron oxide‐based templating leads to the formation of interconnected mesoporous and hollow porous structures, with the potential to embed iron species that impart catalytic functionality. Due to their superior electrical conductivity, such carbons show promising behaviour for applications such as oxidation‐reduction reactions. MnO_2_ offers the unique advantage of morphological control into sheets, rods, wires, and beyond, making the developed porous carbons useful for a myriad of applications, including metal‐ion batteries and pseudocapacitors. Overall, while MgO and ZnO provide fine pore regulation, iron oxides and MnO_2_ are better for designing function‐specific and morphologically diverse porous carbons.

#### 2.5.3 Carbon Yield

Carbon yield is a significant consideration from the cost‐effectiveness perspective. Generally, for porous carbons prepared by chemical activation, such as KOH‐based activation, the yield is ≈10%. With metal oxide templates, it is expected to be higher, as the sole purpose is to create porosity via templating without any carbon burn‐off. For metal oxides, depending upon the reactivity and stability, a general trend in the carbon yield would be MgO>ZnO>Fe_2_O_3_/Fe_3_O_4_ ≈MnO_2_. MgO, with high thermal stability, will act as an inert template, leading to higher yields. ZnO with partial volatilization can lead to slightly reduced yields. Fe_2_O_3_/Fe_3_O_4_ and MnO_2_ are redox‐active templates and can thus decompose carbon during high‐temperature carbonization, leading to lower yields than with MgO or ZnO. In addition to the carbonization behaviour, acid washing significantly controls yield. MgO and ZnO, for instance, can be washed with dilute acids such as HCl, resulting in minimal carbon loss. In contrast, the complete removal of carbonization remnants of Fe_2_O_3_/Fe_3_O_4_ and MnO_2_ may require washing with a stronger acid, resulting in a lower carbon yield.

#### 2.5.4 Ease of Template Removal and Recyclability

MgO and ZnO can be easily etched using dilute acids such as HCl or acetic acid, and both offer the possibilities of regeneration and reuse. The Fe oxide template requires stronger acid washing for removal, and residual Fe species may remain intentionally to harness the positive effects on catalytic activity. MnO_2_ also dissolves in dilute acids but has lower reusability than MgO and ZnO.

Overall, an optimal choice of metal oxide template is crucial for designing the desired porous carbon for a targeted application. Future efforts should focus on hybrid templating strategies (e.g., MgO‐ZnO or MgO‐Fe_2_O_3_ composites) that combine complementary advantages, such as pore architecture control and conductivity, to develop next‐generation porous carbons for various applications. A comparative analysis highlighting the multiple aspects of the different metal oxides used to produce templated porous carbons is summarized in Table 3.

## 3 Synthesis and Application Perspectives of Various Inorganic Metal Salt Nanoparticles

Inorganic metal salts, especially those of sodium and potassium in chloride form, have proven highly versatile templates for the synthesis of porous carbons. Their cubic structure enables the replication of a 3D macroporous type structure in porous carbons. As with other templates mentioned in previous sections, the action of NaCl‐ and KCl‐based templating also extends beyond introducing a porous effect to create short‐range graphitic domains within the structure. The co‐occurrence of porosity and graphitization is well‐suited to electrochemistry‐based applications. A summary of various properties of porous carbons prepared via the NaCl and KCl inorganic salt templating method is provided in **Table** **2**.

**Table 2:** Summary of the various physico‐chemical features of porous carbons obtained using the templating action of inorganic metal salts‐based nanoparticles.

Material	Template/s and carbon precursor/s	Template removal technique	Mechanism of templating/properties of porous carbon	SA [m^2^ g^−1^], PV [cm^3^ g^−1^] and other features	Application attributes	Refs.
NaCl						
Si/C‐122	NaCl nanopore template/ Sucrose	Washing 3 times with DI water and drying at 80 ^0^C	Generation of nanopores by the introduction of NaCl does improve the kinetics processes of lithiation/delithiation.	176 m^2^ g^−1^/4 nm/increase in mesopores with the addition of NaCl crystals	Discharge capacities reach 905 mAh g^−1^, at 0.25 A g^−1^, and 613 mAhg^−1^ at 2 A g^−1^. The total loss in the first 10 cycles is 33%, with a CE of 68.7%.	[109]
3D MPC	NaCl crystallites/ PVP	Water washing	NaCl helps in creating a hierarchical pore structures/ PVP is rich in C&N, and enables homogeneous dispersion, adsorbs on the surface of NaCl crystallites, forming enclosed shells	424.3 m^2^ g^−1^/0.36 cm^3^ g^−1^/ interconnected porous network with large surface area, high graphitization degree, and homogeneous dispersion of Fe/N active sites.	Discharge capacity: ≈900 mAh g⁻¹ (after stabilization), capacity retention: 78% after 100 cycles, First cycle discharge/charge: 1394 mAh g⁻¹ / 957 mAh g⁻¹, First cycle CE: 68.7%	[108]
NCHC	NaCl crystals/ L‐Lysine monohydrochloride (C_6_H_15_O_2_N_2_Cl)	Water washing	NaCl helps improve the surface area, along with producing micropores as well as mesopores / C_6_H_15_O_2_N_2_Cl is a natural amino acid for humans and a naturally available, renewable resource.	347 m^2^ g^−1^, 1.19 nm (micropores) and 8.78 nm (mesopores)/ hollow structure provides a shortened diffusion path between the ions and the electrons. More active sites and a high surface area expose more nitrogen.	ORR: E_onset_ of 0.92 V, with electron transport number of 3.7 and low contribution for the peroxide species, 15%, Tafel slope is 76 mVdec^−1^ with small charge transfer resistance giving high intrinsic catalytic activity. OER: E_onset_ is 90 mVdec^−1^,	[110]
3DAC	NaCl/ Pitch and Phenolic resin	Water washing	NaCl supports in enhancing the surface area, forms interconnected 3D macro as well as mesopores structure, improves the transfer rate between ion, electrode, and electrolyte, and shortens Na+ diffusion pathway/ Low‐cost precursors	32.8 m^2^ g^−1^, 0.074 cm^3^ g^−1^/ 3DAC shows an interlayer distance of 0.408 nm and an I_D_/I_G_ ratio of 1.11.	High reversible capacity (280 mA h g^−1^ at 0.03 A g^−1^), rate capability (66 mA h g^−1^ at 9.6 A g^−1^), high initial CE (75%), and cycling performance (≈188 mA h g^−1^ after 600 cycles at 0.3 A g^−1^).	[111]
a‐EW‐NaCl	NaCl salt/ Egg white	Water washing	NaCl performs as a macropore creating template and also a graphitic catalyst for enhancing the graphitization degree/ Egg white is a highly nutritious food, with gelling, foaming, and emulsifying characteristics, naturally rich in N) and oxygen (O) atoms.	3898 m^2^ g^−1^, 1.81 cm^3^ g^−1^/ Gives an interlayer distance of 0.34 nm, I_D_/I_G_ ratio as 2.25	Specific capacity of 118.8 mA h g^−1^ and capacity retention of 80.1% after 4000 cycles. Lithium‐ion capacitor fabricated with Fe_3_O_4_@C anode, gives energy and power densities of 124.7 Wh kg^−1^ and 16 984 W kg^−1^, respectively, with a capacity retention of 88.3% after 2000 cycles at 5 A g^−1^_._	[112]
FeN/C ‐NH_4_Cl/NaCl	NH_4_Cl‐NaCl/ Poly‐m‐phenylenediamine (p‐mPDA)	Water washing	NaCl improves the nitrogen doping and surface area of the electrocatalyst, providing a 3D framework. Hence, a hierarchical porous structure with dense active sites helps in ORR.	1939 m^2^ g^−1^/ large surface area, and an accessible framework helps in ORR, I_D_/I_G_ ratio is 1.08	In acidic medium, E_onset_ is 0.876 V vs RHE, E_1/2_ is 0.792 V and its Tafel slope: 56 mV dec⁻¹. Electron transfer number is 4.1 (near 4‐electron pathway)	[113]
NaCl‐T	NaCl/ Phenolic resin	Washing with hot, distilled water (80 °C)	Salt‐template cation size affects the surface area and pore size broadening/ Phenolic resin facilitates the formation of micelles, which interact through the hydrogen or covalent bonds and create macromolecular assembly.	1353 m^2^ g^−1^, 0.44 cm^3^ g^−1^/ The mesoporosity provided tunnels for the transport of ions before deep pore penetration, affording a high capacitance retention and I_D_/I_G_ ratio is 0.91	NaCl‐T electrodes give a specific capacitance of 115 F g^−1^ and a capacitive retention of 87% after 10000 cycles	[114]
FeS_2_@G@NS‐3DHCs	NaCl/ Ammonium citrate	Washing with distilled water	NaCl played as a confinement template to inhibit the overgrowth and agglomeration of Fe nanoparticles, supporting the formation of a hollow spherical structure/ Amorphous carbon converted into interlocking stacked graphene–shell	106.50 m^2^ g^−1^, 0.229 cm^3^ g^−1^/ large specific surface areas and an extraordinary amount of mesopores are beneficial for the contact between the electrolyte and the electrode material	Specific capacity (524 mA h g^−1^ at 100 mA g^−1^), rate capability (224 mA h g^−1^ at 8 A g^−1^), and cyclability (99.5% capacity retention over 1000 cycles at 1 A g^−1^).	[116]
SN950 & FePc@SN950	NaCl/ Soybean powder	Stirring 1 hr in water and filtered using a suction pump.	Template promotes mesoporosity, amplifying surface area and facilitating interconnected pore networks, and enhancing charge transfer at the electrode–electrolyte interface / soybean contain natural nitrogen precursors like amino acids, which introduce nitrogen functionalities into the carbon structure	149 m^2^ g^−1^ (SN950)/ Addition of NaCl and increase in pyrolysis temperature enhanced the surface area. The N/C ratio of SN950 is 10.16%.	E_onset_ is 0.97 V and 0.77 V (vs. RHE) in basic and acidic media, respectively, along with a E_1/2_ of 0.92 V and 0.70 V (vs. RHE) under the same conditions. Electron transfer number is 3.5 and 3.8 in basic and acidic media, respectively.	[117]
NPC ‐5	NaCl/ Cationic polyacrylamide	Water washing	The template helps in generating 3D porous structure with numerous macropore / Polyacrylamide exhibit dense block‐like structure, and no obvious pores are observed	499.8 m^2^ g^−1^, 0.5043 cm^3^ g^−1^/ It gives numerous nitrogen (11.38 at%) and oxygen (8.71 at%) containing functional groups.	Specific capacitance of 283.3 Fg^−1^ at 0.5 A g^−1^ in 6 M KOH. Symmetric supercapacitor exhibits an energy density of 34 Wh Kg^−1^ in 1 M ZnSO_4_, while the Zn/ZnSO_4_/NPC‐5 hybrid capacitor delivers a capacity of 115.44 mAh g^−1^, corresponding to an energy density of 85.3 WhKg^−1^.	[115]
HC1500‐10	NaCl/ Petroleum pitch		Petroleum pitch provides desired structures and ultra‐high surface areas, high carbon content, low price, and high aromatic content.	10.4899 m^2^ g^−1^ / SEM images revealed layered structures with a gradual increase in the number of pores, a gradual rise in graphitization, and a decrease in SA with carbonization temperature.	Specific capacity is 430 mAh g^−1^, capacitive retention is 170 mAh g^−1^ after 750 cycles and rate ability is 166 mAh g^−1^ at 2 A g^−1^	[104]
Si@void@C (Pyrrole_2X).	NaCl/ Pyrrole	Water washing	NaCl creates an internal void network. Pyrrole helps in producing carbon layers with a thickness ranging from 5 to 20 nm—carbonization of pyrrole forms disordered and graphitized carbon structures.	The ratio between the D and G was found to be 0.84 for the single‐coated sample and 0.86 for the double‐coated sample.	Double‐coated sample achieves a higher capacity of 670 mAh g^−1^ after 180 cycles.	[106]
CN‐8	NaCl/ Carboxymethyl cellulose	Water washing	NaCl acts as both a template and an inert reaction medium.	1960.07 m^2^ g^−1^, 2.29 cm^3^ g^−1^/ XRD and Raman analysis show turbostratic carbon with both disordered and nanocrystalline graphitic carbon structures.	In a three‐electrode system, HPCM shows a specific capacitance of 414.6 F g^−1^ at 1 A g^−1^ and in a symmetric two‐electrode device, 88.7 F g^−1^ at 1 A g^−1^. The device also showed nearly 100% capacitance retention after 20000 cycles at 30 A g^−1^.	[105]
NC‐32	NaCl/ Gelatin	Water washing	NaCl acts as a solvent and pore‐forming template helps in generating meso and macroporous/ Gelatin is a soluble protein derived from the skin or bone of animals, acting as a source of nitrogen.	2030 m^2^ g^−1^,1.21 cm^3^ g^−1^ / Well‐developed micropores and mesopores.	Specific capacitance of 322 and 217 F g^−1^ is achieved in three‐ electrode and two‐electrode test systems, respectively at 1 A g^−1^. Delivers 195 W kg^−1^ and a specific energy of 7.6 Wh/kg.	[103]
KCl						
WS‐90	KCl/ Walnut shell	Water washing	KCl templating helps in increasing the number of pores and channel structure/ Walnut shell is a good precursor for activated carbon.	1958 m^2^ g^−1^, 1.12 cm^3^ g^−1^/ The longer the activation time, the greater the pore volume and the pore size. K and Cl are wholly removed from the material after the washing.	EDLC with specific capacitance of 245.0 F g^−1^ in 6 molL^−1^ KOH at 0.1 A g^−1^, with capacitance retention ratio of 95.4% at 0.1 A g^−1^ after 4000 cycles	[121]
HPC_2‐4.5_	KCl/ Petroleum asphalt	Water washing	KCl acts as a self‐assembly template and a porogen; it melts and recrystallizes into cubic crystals, leading to the formation of hierarchical porous carbon/ Petroleum asphalt contains polycyclic aromatic hydrocarbons. When heated, it can polymerize and aromatize easily to thin carbon sheets or cages.	3343 m^2^ g^−1^, 1.90 cm^3^ g^−1^/ Surface area as well as the micropore area decreases with KCl dosage. KOH and KCl simultaneously act as activating agents and etch carbon atoms from the skeleton.	HPC shows capacitance of 277 Fg^−1^ at 0.05 A g^−1^, with cyclic stability of 95.1% after 10 000 cycles at 2 A g^−1^ in 6M KOH. In a two‐electrode assembly, specific energy density is 14.2 Wh kg^−1^ at a power density of 445 W kg^−1^ operated in the voltage range of 1.8 V in Na_2_SO_4_ electrolyte	[123]
BiSb@C	KCl/ polyvinylpolypyrrolidone	Water washing several times	The carbon matrix acts as an alleviation agent and suppress the stress/strain of Sb caused by volume change in the electrochemical process.	190 m^2^ g^−1^/ Material shows a mesoporous structure with a pore size of 34 nm. Highly graphitised structure is confirmed from the Raman spectrum (I_G_/I_D_ = 1.1)	KIB anode delivers a reversible capacity of 320 mAh g^−1^ after 600 cycles at 500 mA g^−1^. In full KIBs, coupling with a Prussian Blue cathode provides a capacity of 396 mAh g^−1^ and maintains after 70 cycles.	[124]
CS‐H3	KCl/ Dry wood sawdust	Water washing	KCl prevents carbon from burning and helps in creating an open pore structure/ Wood sawdust has abundant lignocellulosic components, along with KOH, which facilitates effective carbonization and pore formation.	1869 m^2^ g^−1^/ Material shows an open pore structure with interconnected thin walls. With an increase in the KOH ratio, carbon sheets are forming.	Specific capacitance of 286 F g^−1^ in1 Ag^−1^, energy density reaches 5.63 Wh kg^−1^, and power density of 9213 W kg^−1^ in 6 M KOH. It shows a capacitance retention of 99.8% after 10000 cycles.	[125]
KISPC0.8‐4	KCl/ Ginkgo biloba leaf (GBL)	Stirred for 12 hrs, followed by water washing	KCl acts as a template and also as a pore‐forming agent, and it reacts with impurities in the biomass, thus yielding carbon with high purity/ GBL has cellulose, protein, carbohydrates, and a small amount of inorganic minerals, with heteroatoms like (N, S, P).	1675.3 m^2^ g^−1^, 0.88 cm^3^ g^−1^/ Possess abundant micropores <0.6 nm, which act as active sites for accommodating more ions and provide ion‐transport channels.	Specific capacitance of 215.2 Fg^−1^ at 0.05 A g^−1^, capacitance retention of 78.9% and cycling stability with a decay of 1.6% after 10 000 cycles.	[127]
HPCM‐16	KCl/ 2‐hydroxyethyl cellulose (HEC)	Washing with DI water	Molten KCl acts as a sealing medium, which hinders the production of residual volatiles and helps in creating more pores/ HEC is a biocompatible water‐soluble precursor.	1095.74 m^2^ g^−1^, 1.23 cm^3^ g^−1^/ Sponge‐like porous structure with thin carbon sheets having interconnected pores. Surface area and pore volume increased with the addition of KCl	Specific capacitance of 278.09 F g^−1^ at 1 A g^−1^ in 6 M KOH. In two electrodes, the capacitance is 67.23 F g^−1^ at 1 A g^−1^, with a capacitive retention of 80% at 30 A g^−1^. 100% cyclic stability after 10000 cycles	[122]
FBC_10‐cot_	KCl/ Cotton, dandelion, and catkin	Washing with dilute HCl and water	KCl covers the surface of biocarbon, preventing it from air and acts as a fire retardant, helping in producing a 3D hierarchical porous structure with high surface area/ Cotton is a common biomass in daily life, used for producing porous carbon.	1121 m^2^ g^−1^, 0.77 cm^3^ g^−1^/ Pore sizes are generally located in 0.58 nm/3D interconnected pore structure with ultramicropores and defect‐rich material.	EDLC, specific capacitance of 367 Fg^−1^ in 6M KOH. Symmetric supercapacitor gives 93.8 Fg^−1^ at 0.1 A g^−1^ and maintains a columbic efficiency of 100% with a capacitive retention rate of 93.7%	[128]
RKC@600	KCl/ Phthalonitrile (PN) resins,	Water washing	The thermal stability of KCl ensures the structural integrity at high temperature, and also it helps in producing macropores in addition to meso and micropores/ PN has two adjacent cyano groups in a benzene ring, having good thermal, chemical, and mechanical stability	1905 m^2^ g^−1^/ An increase in carbonization temperature increases the interlayer distance. N‐abundant porous carbon rich in micro‐meso and macropores.	Specific capacitance of 300 Fg^−1^ at 1 A g^−1^, and capacitive retention of 96.1% at 10 A g^−1^ after 10, 000 cycles	[157]
K3K5	KCl/ Macadamia nuts	Washing with HCl and DI water	KCl increases the volume of narrow micropores and average pore size, which results in a hierarchical porous structure/ Macadamia nuts are a high‐value crop grown all over the world.	1046.5 m^2^ g^−1^, 0.47 cm^3^ g^−1^/ KCl activation promotes surface area by 50.7% and the pore content by 54.5%.	EDLC with a specific capacitance of 273.5 Fg^−1^ at 0.5 A g^−1^, CO_2_ adsorption at 0 °C and 25 °C as 6.48 and 4.11 mmolg^−1^.	[129]
LR‐KPC‐4	KCl/ Coal liquefaction residue (CLR)	Washing with 3 M HCl and DI water	KCl promotes the KOH activation and increases the micro‐mesopores in the carbon structure, and also acts as an auxiliary template/ CLR is a byproduct of coal direct liquefaction; the heavy oil and asphaltene can be easily graphitised.	2542 m^2^ g^−1^, 1.124 cm^3^ g^−1^/ Well‐developed porous structure with high surface area	Specific capacitance of 375 Fg^−1^ at 0.5 A g^−1^ and superior rate capacity. Symmetric supercapacitor shows specific capacitance of 256 Fg^−1^ at 0.5 A g^−1^, energy density of 8.9 Wh kg^−1^ at 125 W kg^−1^.	[126]
NHPC132	KCl/ Rice husk pyrolysis bio‐oil	Washing with 2M HCl and water	KCl acts both as the molten phase and built‐in template/ Heavy oil is abundant in benzene rings and carbon content, with an ideal pore structure	2388 m^2^ g^−1^ / Interconnected and hierarchical pore structure with random orientation of graphitic layers.	Specific capacitance of 405 Fg^−1^ at 0.5 A g^−1^ with a high retention of 263 Fg^−1^ at 50 A g^−1^. In a two‐electrode, high energy density of 17.90 Wh kg^−1^ at a power density of 300 W kg‐1.	[158]
HCPC‐1‐1‐800‐IMP	KCl/ Corncob powder	Water washing	KCl improves the pore structure.	1914 m^2^ g^−1^ and 0.96 cm^3^ g^−1^ / Exhibited high specific surface area and hierarchical pore structure, and the ratio of volume mesopore and macropore to micropore was ≈1:2.2.	Specific capacitance of 72.7 Fg^−1^ at 1 A g^−1^ and a high energy density of 22.7 Wh kg^−1^ at 750 W kg^−1^. After 5000 cycles at 5 A g^−1^, the CE remained close to 100% and the specific capacitance retention rate was 93.6%.	[130]

**SA** – Surface area, **PV**‐ Pore volume; **ORR**‐Oxygen reduction reaction, **OER** – Oxygen evolution reaction, **EDLC** – Electric Double layer Capacitance, **F g^−1^** – Faraday per gram, **A g^−1^** – Ampere per gram, **mmolg^−1^** = millimole per gram; **Si/C‐122** – SiO_2_ nanoparticles in porous carbon matrix with a weight ratio of precursor as Si:Sucrose:NaCl as 1:2:2; **3D MPC** – Three dimensional macroporous carbon materials derived from PVP, template with NaCl and heated at 900 °C, PVP‐ polyvinylpyrrolidone; **NCHC**‐ Nitrogen doped carbon hollow tube from biomass l‐lysine monohydrochloride using NaCl template heated at 1000 °C; **3DAC** – 3 D amorphous carbon derived from phenolic resin synthesized via a facile NaCl template‐assisted method carbonized at 1100 °C; **a‐EW‐NaCl** – Activated carbon powder derived from egg white using NaCl as the template annealed at 600 °C; **FeN/C‐ NH_4_Cl/NaCl** – Iron, nitrogen co‐dopped in carbon catalyst using NH_4_Cl and NaCl mixture as template; **NaCl‐T** – Carbon material synthesized from phloroglucinol (1,3,5‐benzenetriol) using NaCl as template and Pluronic F127 as soft template, T represents the soft template; **FeS_2_@G@NS‐3DHCs** – Core‐shelled FeS2@graphene is uniformly encapsulated in the N/S co‐doped 3D hollow carbon sphere; **FePc** – Iron phthalocyanine, SN950‐ Soybean powder carbonized at 950 °C, **FePc@SN950**; Iron phthalocyanine immobilized on the soybean‐derived carbon carbonized at 950 °C; **NPC ‐5** – N/O co doped porous carbon derived from cationic polyacrylamide with 5 g of NaCl; **HC1500‐10** – Hard carbon derived from petroleum pitch heated at 1500 °C for 10 hrs; **Si@void@C (Pyrrole_2X) –** Silicon mixed with NaCl double coated with pyrrole (2X denotes the number of carbon coating); **CN‐8** – Hierarchically porous carbon materials derived from carboxymethyl cellulose (CMC), with CMC: NaCl as 1:8; **NC‐32** – Porous carbon derived from gelatin with NaCl and Caustic soda mass ratio as 3:2; **WS‐90** – KCl templated porous carbon derived from walnut shell, heated in tubular furnace at 900 °C for 90 min; **HPC_2‐4.5_** – Hierarchical porous carbon derived from petroleum asphalt template by KCl with 2 g of petroleum asphalt and 4.5 g of KCl; **BiSb@C** – Bismuth−antimony alloy nanoparticles embedded in a porous carbon matrix; **CS‐H3** – Porous carbon derived from dry saw wood heated at 800 °C with 3 g of KOH; **KISPC_0.8‐4_**_–_ N/S‐co‐doped thin‐sheet porous carbon (KISPC) from GBL using molten salt KCl/K_2_CO_3_ as template and activation agents, 0.8 is the molar ratio of K_2_CO_3_ and 4 is the molar ratio of precarbonised powder; **HPCM‐16** – Hierarchically porous carbon materials (HPCMs) derived from 2‐hydroxyethyl cellulose (HEC) carbonized at 1000 °C with 16 g of KCl; **FBC_10‐Cot_**_–_ Hierarchical porous carbon derived from cotton biomass by flame burning carbonization technique for 10 min burning; **RKC@600** – Porous carbon from 1,3‐bis(3,4‐dicyanophenoxy)benzene carbonized at 600 °C with KCl as the byproduct; **K3K5** – Hierarchical porous carbon derived from Macadamia nuts heated at 800 °C using K_2_C_2_O_4_.H_2_O as activator and KCl as salt template, 3 g of K_2_C_2_O_4_.H_2_O and 5 g of KCl; **LR‐KPC‐4** – Porous carbon derived from CLR by one step activation using KCl assisted KOH activation at 800 °C (taking 1g of LR, 2 g of KOH and 4 g of KCl); **NHPC132** – N doped porous carbon derived from rice husk‐derived heavy bio‐oil with egg shell as template and KCl‐KHCO_3_ simultaneous activators, activated by taking 1g of carbonized sample, 3 g of KCl and 2g of KHCO_3_; **HCPC‐1‐1‐800‐IMP** – hierarchical porous carbon derived from corncob hydrochar (HCPC) was prepared by KCl molten salt enhancing K_2_CO_3_ activation, taking 1 as the mass ratio between K_2_CO_3_ and HC and 1 mass ratio between KCl and HC, activated at 800 °C taking pre‐mixing method as impregnation.

### 3.1 NaCl

**Figure 10:**
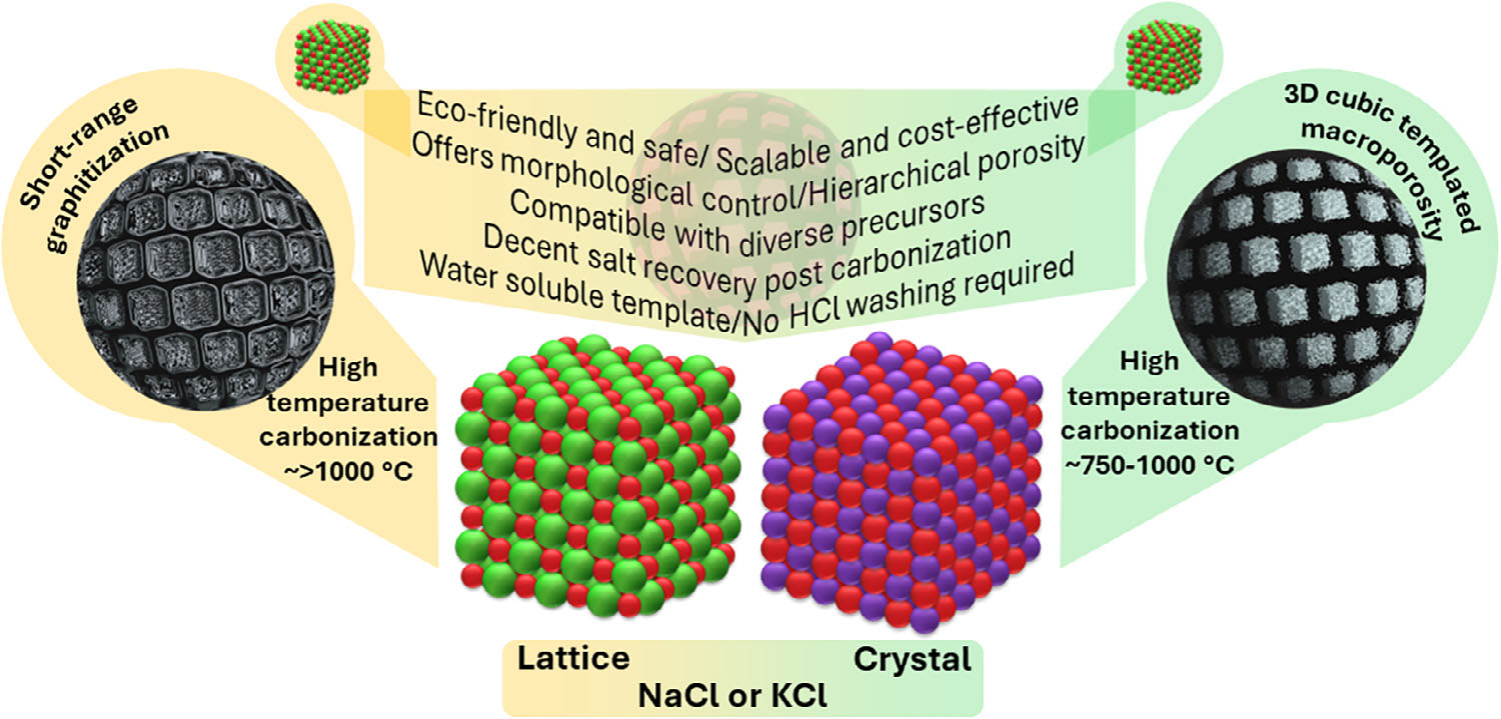
A representation of the various features of the NaCl and KCl‐based templating process for the synthesis of porous carbons.

NaCl, an abundant and inexpensive inorganic salt, makes an ideal sacrificial template for creating macroporosity in carbons.^[^ ^103^ ^]^ The porosity domains could also span the micro and meso scales, and a small degree of graphitization can be introduced, depending on the choice of carbonization temperature.^[^ ^104^ ^]^ It is thermally stable, with a melting point of 801 °C, and can be used as a template that does not chemically react with carbon precursors, thereby generating no unwanted by‐products. Like other inorganic templates, it can be removed by a dilute acid wash after carbonization to eliminate carbonization remnants from the pores.^[^ ^105^ ^]^ However, due to its high solubility, any unreacted NaCl after carbonization can also be removed by water washing alone, offering a combination of environmental and economic advantages.^[^ ^106^ ^]^ Most studies report that washing with water after carbonization is an effective way to remove unwanted residues from the materials.^[^ ^107^ ^]^ While the primary role of NaCl is to act as a template to create a 3D framework in porous carbons during carbonization, its ability to melt at high temperatures and form eutectic mixtures with other salts can enhance its reactivity with carbon precursors.^[^ ^108^ ^]^ It is also possible to vary the size and shape of NaCl crystals through recrystallization, thereby providing better control over the porous carbon framework. Finally, it is compatible with almost any carbon precursor, including biomass, nitrogen‐rich compounds, MOFs, and polymers. Numerous studies have reported the use of NaCl alone as a sacrificial template or in combination with other salts to form eutectic salt mixtures, thereby creating the desired type of porosity in carbon‐based materials. This approach can be applied to develop materials suited across a diverse range of application domains. A visual summary of the features of NaCl‐based templating for the fabrication of porous carbons is shown in **Figure** **10**.

**Figure 11:**
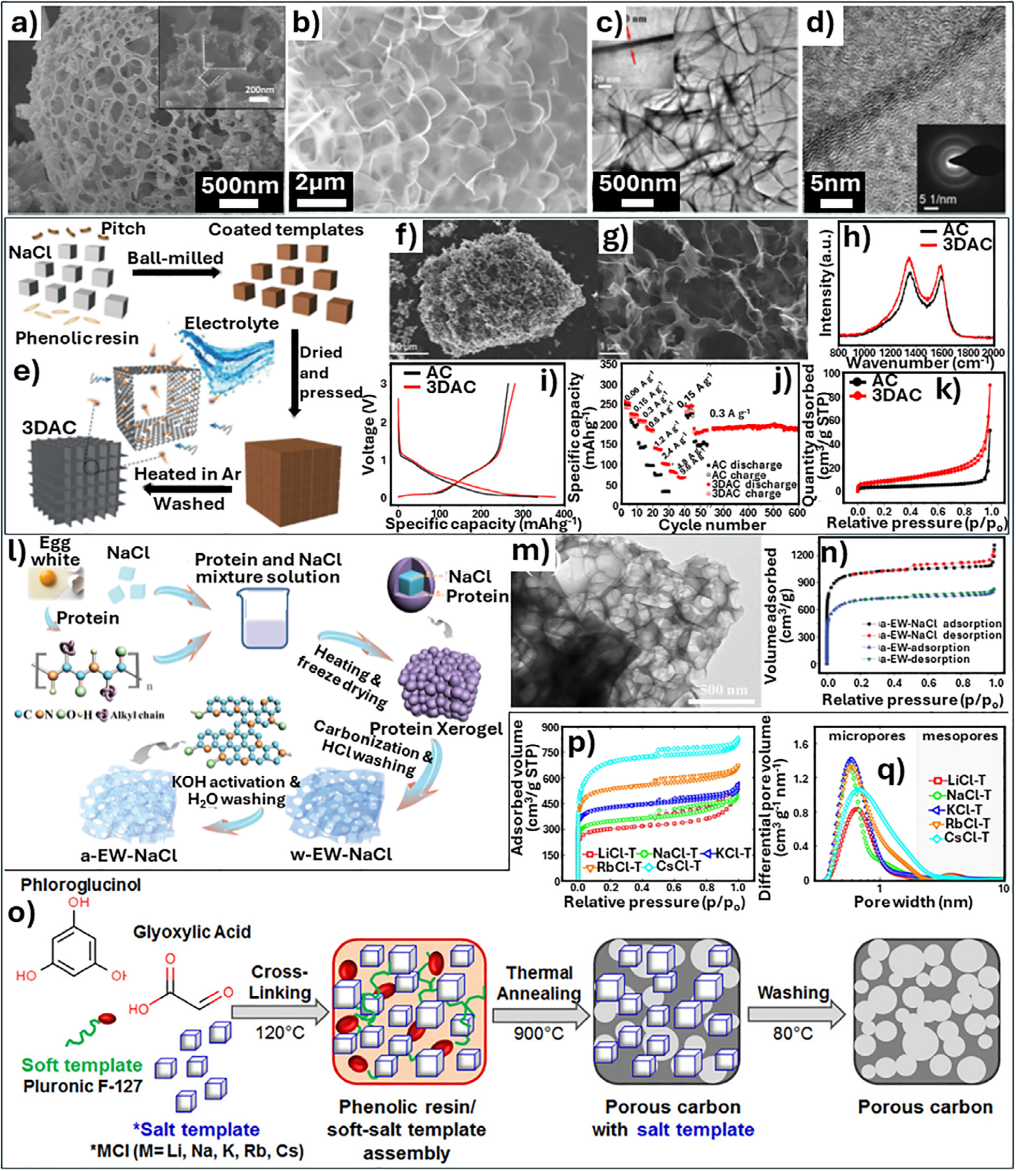
a) Cubic‐shaped macropores created via NaCl in porous Si/Carbon composite, Reproduced with permission^[^ ^109^ ^]^ Copyright Elsevier 2016, b–d) SEM, TEM, and HRTEM images of N‐doped carbon hollow cubes via the NaCl templating effect showing the micron‐size voids, 10 nm wall thickness, and particle graphitization induced by NaCl templating, Reproduced with permission^[^ ^110^ ^]^ Copyright Royal Society of Chemistry 2017, e–k) synthesis schematic of a 3D amorphous carbon using pitch, phenolic resin, and NaCl template, SEM images depicting its macroporosity, N_2_ sorption, and Raman showing its superiority with the non‐templated amorphous carbon (AC), galvanostatic first charge discharge (CD) profile, and rate capability at various current densities, Reproduced with permission^[^ ^111^ ^]^ Copyright Wiley 2018, l–n) egg white based porous carbon synthesized via the dual approach of NaCl templating and KOH activation, cubic shaped topography as per HRTEM imaging and the N_2_ sorption isotherm curves of materials synthesized with and without NaCl templating, Reproduced with permission^[^ ^112^ ^]^ Copyright Royal Society of Chemistry 2018, and o–q) synthesis schematic of porous carbon via a combined effect of soft templating and salt templating using different alkali metal salts, their nitrogen sorption and pore size distribution curves, Reproduced with permission^[^ ^114^ ^]^ Copyright American Chemical Society 2021.

Cubic‐shaped pores are often associated with the use of a NaCl template, which is attributed to its cubic crystal structure. Although, in general, the use of NaCl crystals will produce voids or macropores (≈500 nm in size), as demonstrated in a study based on Si/C carbon composites (**Figure** **11**a), it can also influence the high‐temperature carbonization of carbon‐containing precursors and induce the formation of mesoporous structures.^[^ ^109^ ^]^ The size of the voids can be extended to even 1–2 µm with a wall thickness of 10 nm via the NaCl templating effect, when utilized in conjunction with specific precursors such as L‐lysine monohydrochloride, an amino acid‐based salt, at a carbonization temperature of 1000 °C (Figure 11b,c).^[^ ^110^ ^]^ Although the main exciting feature of this work is the production of micro‐sized hollow carbon spheres, NaCl also promoted the development of short‐range graphitic domains in the carbon structure (Figure 11d) and a pore size mix in micro (1.19 nm) and meso (8.78 nm) domains. A combination of factors, including the inherent N doping in lysine, high surface area (347 m^2^ g^−1^), partial graphitization, exposure of pyridinic and graphitic nitrogen, and a minor charge‐transfer resistance, proved beneficial for achieving activity comparable to that of commercial 20 wt% Pt/C or RuO_2_. High temperatures of ≈1000 °C are commonly used to maximize the templating effect of NaCl. Lu et al. in 2018 used a two‐step synthesis temperature wherein a pitch and phenolic resin were carbonized together along with NaCl first at 750 °C, with the carbonized materials being washed to eliminate any NaCl, dried, and then followed by a second carbonization step at 1100 °C to maximize the conductivity, graphitization and fully develop the macroporosity in a 3D amorphous carbon (3DAC) (Figure 11e).^[^ ^111^ ^]^ Quality‐wise, the 3DAC was much superior to its counterpart, amorphous carbon (AC) synthesized without a NaCl template (Figure 11f–i). Micron‐sized (5–40 µm) particles with a 3D amorphous open structure, partial graphitization, a mix of micro, meso, and macropores yielded a reversible capacity of 280 mAh g^−1^/0.03A g^−1^ and rate capability of 66 mAh g^−1^/9.6 A g^−1^ for a SIB (Figure 11j‐k).

NaCl‐templated porous carbon with large macropores generally has a low surface area, which could be a limiting factor from several application perspectives. One of the typical and widely adopted methods to improve the surface area is to include an additional step of chemical activation with KOH after the templating process. For instance, the egg white based porous carbons (a‐EW‐NaCl) yielded an impressively high surface area of 3898 m^2^ g^−1,^ which was obtained after chemical activation of the macroporous NaCl templated porous carbon formed in the initial step (Figure 11l,m).^[^ ^112^ ^]^ This increase in surface area was significantly higher than that of a porous carbon, wherein no NaCl templating effect but only KOH activation was carried out (a‐EW – ≈2714 m^2^ g^−1^). The higher sorption of N_2_ (Figure 11n) also signifies a concurrent increase in pore volume from 1.26 to 1.81 cm^3^ g^−1^. Furthermore, an increase from 2.25 to 2.46 in the I_D_/I_G_ ratio in Raman spectral peaks signifies the growth of short‐range crystalline domains caused by the NaCl templating effect. From an application perspective, the materials delivered a good capacity of 118.8 mA h g^−1^ at 0.1 A g^−1^ in LICs. Later, Hou et al. (2020) proposed that NaCl templating can also be used in conjunction with NH_4_Cl; however, the surface area was only 1939 m^2^ g^−1.[^ ^113^ ^]^ Salt templating using NaCl and other salts in the same group can also be combined with a soft template, such as Pluronic F127, for porous carbon derived from a phenolic resin.^[^ ^114^ ^]^ However, an additional step is required to establish polymerization of the precursor into the resin at 120 °C for 6 h before proceeding to carbonization at 900 °C for 1 h (Figure 11o). It was also observed from N_2_ sorption results that, although the materials displayed a microporous nature, salts with smaller cations, such as lithium and sodium, produced a higher number of mesopores (Figure 11p,q) and greater graphitization of the carbon than salts with larger cations, such as potassium, rubidium, or cesium. A significant aspect of this study was the recovery of unused chloride salt after carbonization, which exceeded 80% for all salts. This finding leads us to conclude that these inexpensive inorganic templates can be reutilized, offering both economic and environmental benefits. Further confirmation of the recovery and reuse of NaCl as a template came from Song et al. (2024), who found that after its templating action on polyacrylamide at 700 °C, the carbonized product can be washed with water, and the obtained solution can be dried to collect recycled NaCl.^[^ ^115^ ^]^ Thereafter, it was reused with polyacrylamide for further carbonization, and the resulting material showed a specific capacitance performance similar to that of the original.

**Figure 12:**
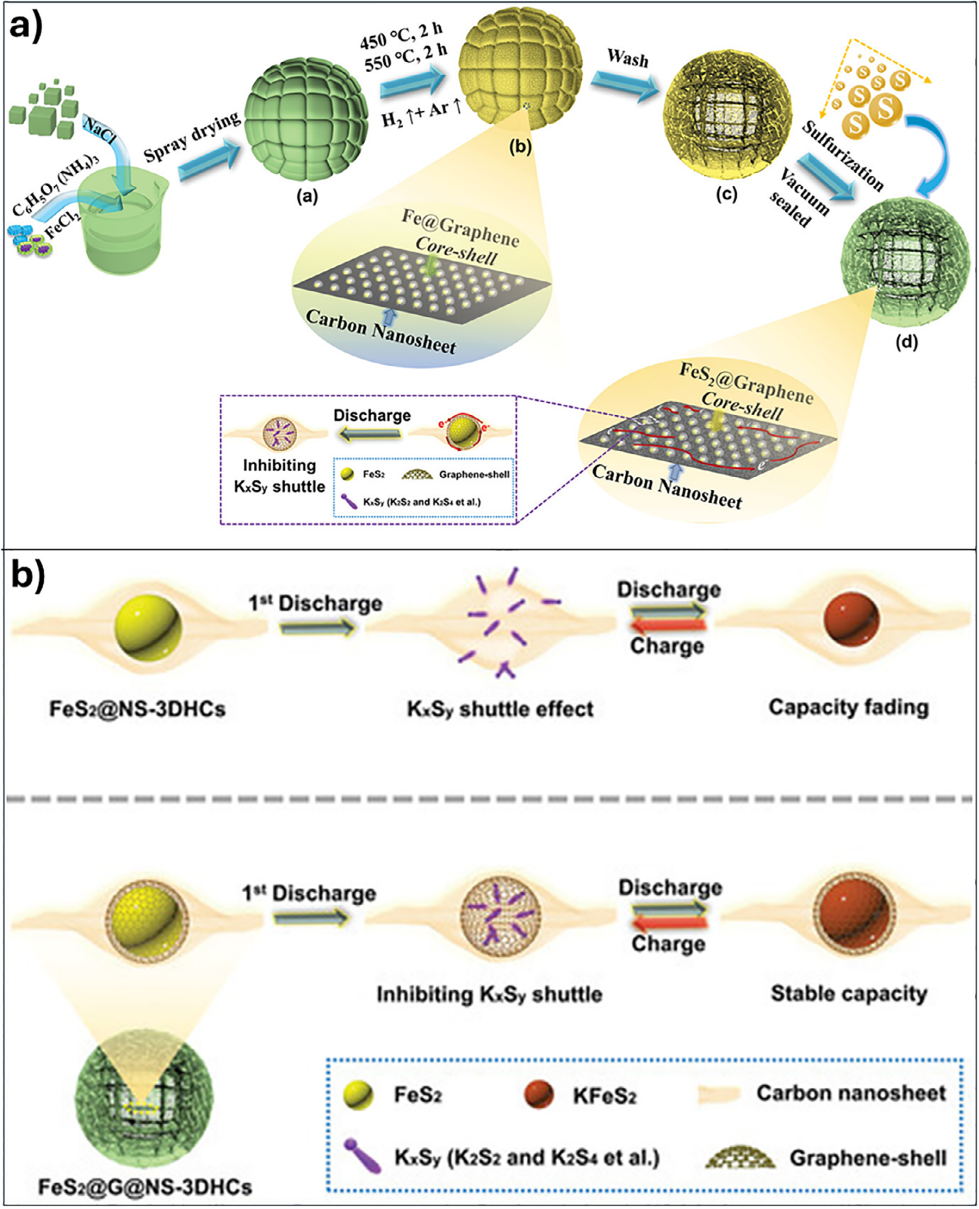
a) Synthesis schematic of FeS_2_ encapsulated with graphitic domains of a 3D hollow carbon sphere, and b) mechanistic illustration of how a pocket‐like graphitic shell loaded with FeS_2_ controls the shuttling effect of potassium, polysulfides, Reproduced with permission^[^ ^116^ ^]^ Copyright Wiley 2022.

Realizing that NaCl can produce 3D macroporous structure along with short‐range graphitic domains in carbons, Chen et al. (2022) utilized this strategy to in situ generate FeS_2_ nanoparticles confined within the graphitized matrix of 3D hollow carbon spheres.^[^ ^116^ ^]^ First, ammonium citrate and ferrous chloride were mixed with NaCl, and the mixture was spray‐dried, then reduced at 450 °C and carbonized at 550 °C to obtain nitrogen‐doped 3D hollow carbon spheres (Fe@N‐3DHCs). The Fe particles were transformed into FeS_2_ via reaction of Fe@N‐3DHCs with sublimed sulfur in consecutive heating steps at 400 and 300 °C to obtain graphene–shell‐encapsulated FeS_2_ embedded in N/S codoped 3D hollow carbon spheres (FeS_2_@G@NS‐3DHCs) (**Figure** **12**a). From an application point of view, this composite not only proved to be a meticulous structural platform to deliver a specific capacity of 524 mAh g^−1^ at 0.1 A g^−1^ in KIBs, but it also plays a vital role in inhibiting the shuttling of the potassium polysulfides during charging and discharging operations (Figure 12b). Overall, this is an interesting approach and can be extended to other carbon‐based precursors and other metal sulfides and related species for suitability in various specific applications.

Another fascinating aspect of using NaCl as a template is harnessing its ability to prevent nitrogen vaporization at higher temperatures, thereby creating porous carbons doped with appreciable amounts of nitrogen. Rathor et al. (2024) confirmed this by utilizing soybean as a starting precursor source of carbon and nitrogen and producing porous carbons via NaCl‐based templating.^[^ ^117^ ^]^ The porous carbons could retain 11.09% nitrogen in the porous carbon matrix at a carbonization temperature of 850 °C, which is a significant amount. It is reasonable to note here that during carbonization, volatiles, primarily composed of oxygen, nitrogen, and hydrogen, escape through the carbon and create porosity. In contrast, NaCl, a stable inorganic salt, merely exerts a templating effect because it does not react chemically with carbon or other elements. This contrasts with porous carbons synthesized by chemical activation, for instance with KOH, as KOH can chemically react via various redox pathways to enhance the elimination of carbon and heteroatoms, such as nitrogen and oxygen.^[^ ^118^, ^119^, ^120^ ^]^ So, there must be a weigh‐off between chemical activation with KOH and templating with NaCl, as the former produces a high surface area with lower heteroatom content, whereas the latter can produce the opposite results.

To sum up, NaCl has proved to be an excellent inorganic salt‐based templating approach, which is not only economically and environmentally beneficial, but the produced porous carbons possess reasonable porosity characteristics to attain competitive applications performance as compared to some of the conventional methods of porosity creation in carbon‐based materials, including chemical activation or silica‐based templating.

### 3.2 KCl Templating

**Figure 13:**
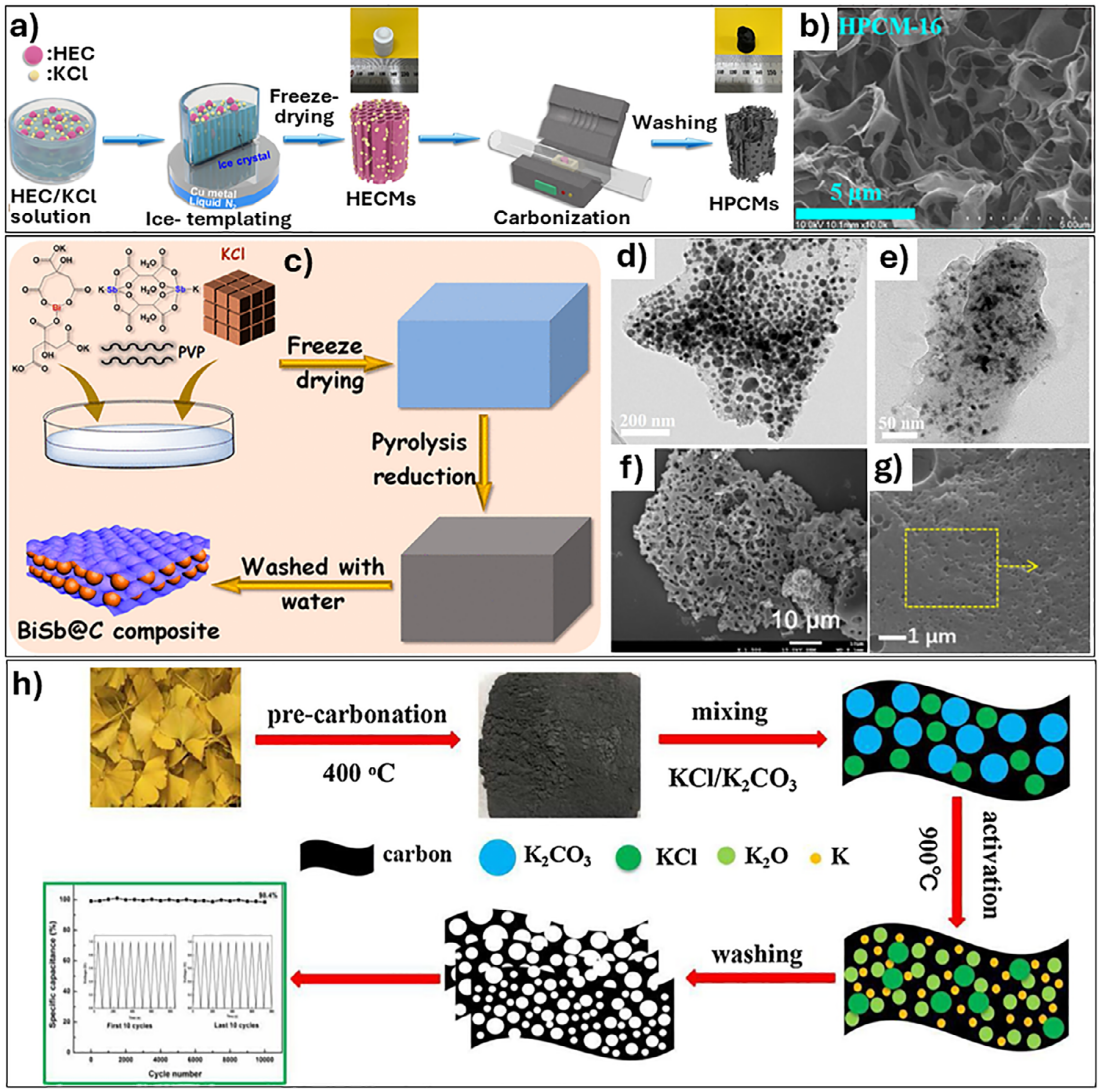
a–b) Synthesis schematic of the porous carbon derived from 2‐hydrocycellulose (HEC) and KCl templating, and the FE‐SEM image of the material HPCM‐16, which was synthesized with the highest amount of KCl template usage, Reproduced with permission^[^ ^122^ ^]^ Copyright Elsevier 2022, c–e) synthesis schematic of the bismuth and antimony alloy nanoparticles support on porous carbon matrix (BiSb@C) via KCl templating pathway, and its TEM images before electrochemical measurement and post 450 cycles stability testing, showing the sturdiness of the material, Reproduced with permission^[^ ^124^ ^]^ Copyright American Chemical Society 2020, f,g) SEM images of CS‐H3, a material derived from dry wood sawdust through KCl templating and KOH activation, Reproduced with permission, Reproduced with permission^[^ ^125^ ^]^ Copyright Elsevier 2020, SEM image of LR‐KPC‐4 synthesized via KCl templating and KOH activation of coal liquefaction residue (CLR), Reproduced with permission^[^ ^126^ ^]^ Copyright Elsevier 2024, and h) synthesis schematic of N, S‐codoped hierarchically porous carbon nanosheets using KCL templating and K_2_CO_3_‐based chemical activation of ginkgo biloba leaves, Reproduced with permission^[^ ^127^ ^]^ Copyright Elsevier 2021.

Like NaCl, KCl is another inorganic hard templating choice for porous carbons. It also has a face cubic crystal domain, which can lead to the formation of cubic voids in the porous carbons, depending upon the quantity employed. In general, even in NaCl templating studies, the larger amounts of these two templates, in multiples of 5–10 relative to the starting carbon precursor, produce large cubic voids, which may not be possible with smaller quantities. There are only subtle changes in the structure and properties of KCl as compared to NaCl. Its lattice parameter is 0.629 nm (NaCl – 0.564 nm), melting point is 770 °C (NaCl – 801 °C), solubility in water is 34g/100 mL (NaCl‐35.9g/100 mL), and ionic radius of K^+^ is 138 pm (Na^+^ – 102 pm). These attributes imply that KCl can withstand high temperatures, produce slightly larger voids compared to NaCl, and can be easily washed off with water after carbonization. In 2018, Cao et al. explored walnut shells for KCl templating, in which 50 g of precursor was reacted with 50 g of KCl at 900 °C under inert conditions, and the excess KCL and carbonization remnants were washed away with warm and cold water washings.^[^ ^121^ ^]^ A reasonable surface area of 929 m^2^ g^−1^ with a pore volume of 0.51 cm^3^ g^−1^, mostly concentrated in micropores, was observed. The KCl templating step was followed by CO_2_‐based physical activation, which increased the porous texture of the materials by almost two‐fold. This study is a perfect example that KCl‐based templating can be employed for the large‐scale synthesis of porous carbons, beyond the conventional laboratory‐based synthesis of 1g or 2g of the materials. Another instance highlights that the KCl‐based ice‐templating technique is an effective approach for deriving porous carbons with well‐developed porous texture from 2‐hydroxycellulose (HEC).^[^ ^122^ ^]^ After infusing KCl in HEC, the mixture was frozen using liquid N_2_ and then freeze‐dried to obtain HEC/KCl monoliths, which were carbonized at 1000 °C to yield hierarchically porous carbon materials (HPCMs) (**Figure** **13**a). Water washing was adopted to remove any residues left from carbonization, and it was observed that a higher amount of KCl resulted in a lower yield of porous carbon. The lower yield was associated with the material (HPCM‐16) with the highest surface area (1095.74 m^2^ g^−1^) and pore volume (1.23 cm^3^ g^−1^), which exhibited a 3D sponge‐like morphology and thin carbon walls (Figure 13b). These attributes led to a superior specific capacitance of 278.09 F g^−1^ at 1 A g^−1^ in 6 M KOH for a three‐electrode supercapacitor and 67.23 F g^−1^ at 1 A g^−1^ for a two‐electrode supercapacitor. This study is a good example of creating porosity in porous carbons and achieving promising electrochemical results using the water‐soluble salt KCl, without resorting to expensive silica templating or aggressive KOH‐based chemical activation. For the sake of comparison, although petroleum asphalt‐based HPC produced using a combined effect of KCl templating and KOH activation delivered a surface area of 3580 m^2^ g^−1^, the specific capacitance reached only 277 F g^−1^ at a much lower current density of 0.05 A g^−1^ in 6M KOH.^[^ ^123^ ^]^ Freeze drying has been a common approach with the use of KCl as a template and was again used by Xiong et al. (2020) to fabricate an alloy nanoparticle of bismuth (Bi) and antimony (Sb) supported on a porous carbon matrix (BiSb@C) for utilization in KIBs (Figure 13c).^[^ ^124^ ^]^ Bismuth potassium citrate and potassium antimonyl tartrate sesquihydrate supplied Bi and Sb, and the carbon from the organic ligand parts and KCl delivered the templating effect. Bi and Sb alloy nanoparticles were uniformly distributed in the carbon matrix, as observed by TEM, and the structure did not undergo any noticeable change after 450 cycles of continuous electrochemical testing (Figures 13d,e). From an application perspective, BiSb@C delivered a good reversible capacity of 598.2 mAh g^−1^ at 0.1 A g^−1^, 70.2% coulombic efficiency, and 97.5% capacity retention after 100 cycles at 0.5 mAh g^−1^. KCl templating, in conjunction with other K‐salt‐based chemical activation, has been widely reported. The chemical activation step is introduced to enhance porosity and surface area, which are otherwise low in KCl‐templated carbons. However, as discussed earlier, it may not necessarily improve the application attributes of the materials compared to using a standalone KCl template. One instance report that 3 g of dry sawdust with five times the amount of KCl and an equal amount of KOH equates to a surface area of 1869 m^2^ g^−1^ in the developed porous carbon, CS‐H3 (Figure 13f), and the material delivered a specific capacitance of 286 F g^−1^/1A g^−1^, energy and power densities of 5.63 Wh kg^−1^ and 9213 Wkg^−1^ in 6 M KOH as an electrolyte.^[^ ^125^ ^]^ In comparison, the carbonization of a combination of 1 g of a coal liquefaction residue, 4 g of KCl, and 2 g of KOH (LR‐KPC‐4/Figure 13g) produced a surface area of 2542 m^2^ g^−1^ and a large pore volume of 1.124 cm^3^ g^−1^, which results in a specific capacitance of 375 F g^−1^ at 0.5 A g^−1.^ ^126^ Although both studies employed a carbonization temperature of 800 °C and different precursor compositions, the key factors that led to materials with different porosities were washing with 3M HCl and the use of a carbon‐rich precursor, which is ideal for KOH to react in the second study. Considerable changes can occur when the activation agent is changed as well. For instance, KCl and K_2_CO_3_, a well‐explored combination of templating and chemical activation, can produce N, S co‐doped porous carbons from ginkgo biloba leaves (GBL) with interconnected sheetlike structures and a surface area of 2130.4 m^2^ g^−1^ (Figure 13h).^[^ ^127^ ^]^ Application‐wise, a specific capacitance of 215.2 F g^−1^ at 0.05 A g^−1^, with a 78.9% retention at 20 A g^−1^ and only 1.6% capacity decay after 10 000 charge/discharge cycles was observed. In other instances, the combination of KCl and K_2_CO_3_ produced porous carbons from cotton with a surface area with the extreme of 1509 m^2^ g^−1^ and a specific capacitance of 367 F g^−1^ in 6M KOH,^[^ ^128^ ^]^ KCl and K_2_C_2_O_4_ mediated porous carbon derived from macadamia shell, showed predominantly mesopores and narrow micropores which supported the delivery of 6.48 mmol g^−1^ of CO_2_ and 279.3 F g^−1^ specific capacitance.^[^ ^129^ ^]^ On the other hand, KCl and K_2_CO_3_ with a combined action of corncob hydrochar produce porous carbons with a surface area of 1914 m^2^ g^−1^ and achieve a specific capacitance of 255.4 F g^−1^ at 0.5 A g^−1.[^ ^130^ ^]^ Overall, KCl templating is a good approach to infuse porosity into porous carbons, which reside mostly in mesopores and to a lesser extent in micropores. Its use for this purpose can be established by either the salt alone or in combination with activating agents.

### 3.3 Comparative Insights Among NaCl and KCl Templates

Both NaCl and KCl have been successfully deployed as templates and share the common advantages of being simple, low‐cost, and environmentally benign for constructing porous carbons. Although their chemical properties and crystalline structures are closely similar, subtle differences in various physical and thermochemical properties result in slightly different outcomes for templated porous carbons. As mentioned earlier, the larger lattice parameter of KCl could induce the formation of marginally larger voids than NaCl. The structural rigidity of the porous carbons can also vary slightly, as NaCl, with a higher melting point, can provide a more rigid template than KCl. NaCl generally produces a 3D cubic macroporous structure with some mesopores, while KCl induces the formation of mainly mesopores rather than strictly macroporous domains. In terms of carbon yield, NaCl performs better than KCl because it acts as a physical spacer and does not react chemically with the precursor. The inertness of NaCl is also favourable for preserving heteroatoms, such as nitrogen, in the carbon structure. The washing of the templates after high‐temperature carbonization also varies slightly. NaCl, with marginally better water solubility, offers greater ease of water washing than KCl. Water washing with NaCl has been demonstrated on numerous occasions. This renders both templates more sustainable for recovery and reuse than metal oxides. From an application perspective, NaCl‐templated porous carbons, owing to their 3D macro‐mesoporous framework and partial graphitization, can perform well as battery anodes and catalyst supports, whereas KCl‐templated porous carbons, benefiting from their interconnected mesoporous texture, are well suited for supercapacitors, adsorption, and electrocatalysis. The porous carbons derived from either template also provide a platform for further chemical activation, thereby enabling complementary enhancement of application performance.

### 3.4 Composite Salt Templates

Composite salts can exhibit eutectic behavior, which stands for a lowered melting point of the salt mixture as compared to individual salts during carbonization. For instance, NaCl melts at ≈801 °C and KCl melts at ≈770 °C, but their eutectic mixture has a lower melting point of ≈655–670 °C. This is useful for carrying out carbonization at lower temperatures, which could be a significant energy‐saving factor in large‐scale synthesis operations. Moreover, the salt mixture can penetrate or infiltrate the carbon precursor more efficiently to create interconnected meso and macropores. Furthermore, the different crystal sizes of salts allow for improved particle packing and the development of a controlled morphology. Various combinations, including NaCl and KCl, NaCl and ZnCl_2_, KCl and CaCl_2_, and others, have been explored for this purpose. Perhaps the research has also extended to ternary salt composites. In addition to the advantages described above, the multi‐templating method is also low‐cost and easily scalable. It can be applied to a versatile range of precursors, including biomass, polymers, resins, and other similar materials. Washing excess salt with water is always the main attraction of salt templating, and it is also found in the composite salt templating method. The only downside is that this template primarily produces meso‐ and macropores, while micropores must be created separately via chemical treatment with KOH and related chemicals.

**Figure 14:**
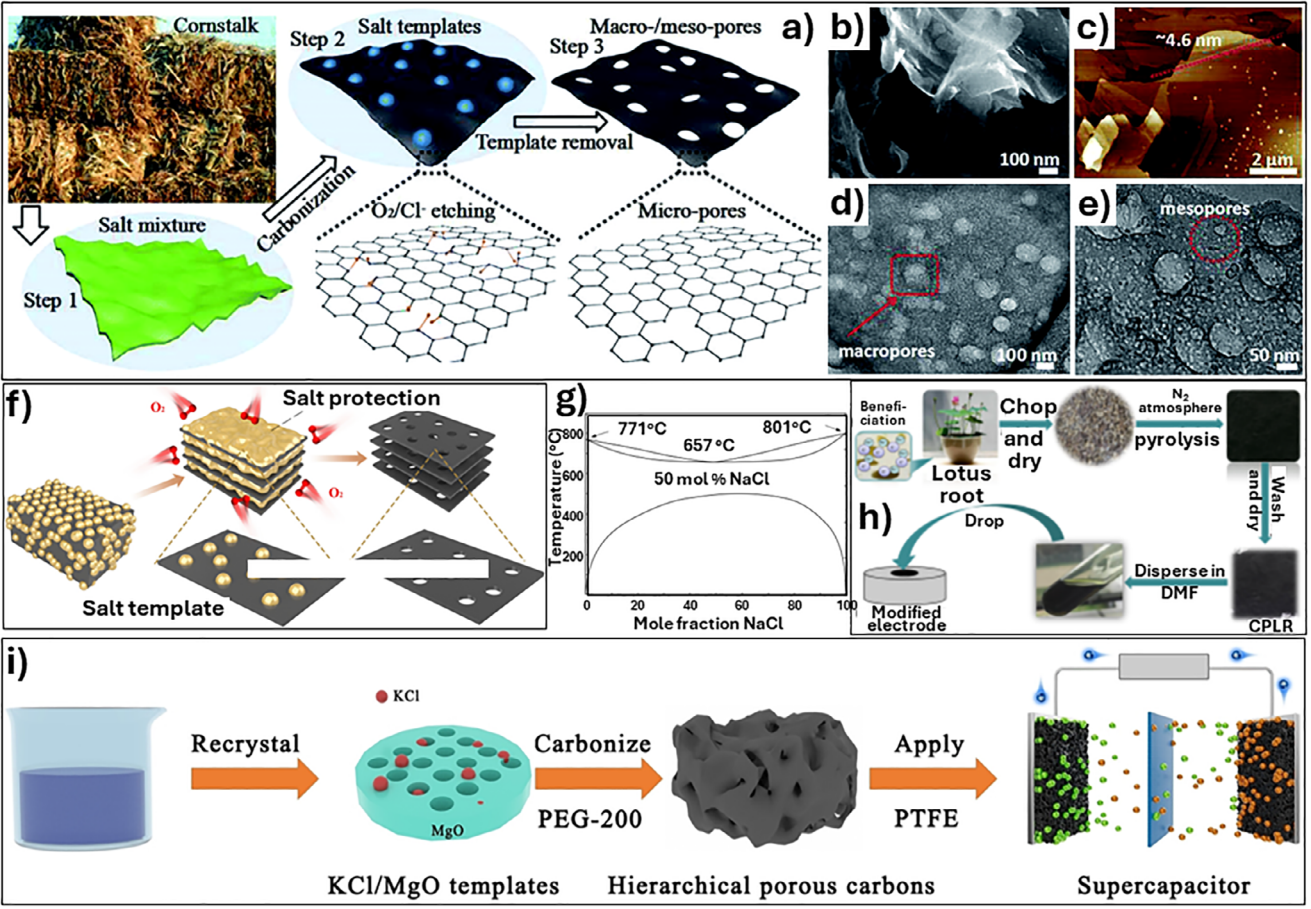
a–e) Synthesis schematic of porous carbon sheets from cornstalk using the dual salt NaCl and KCl as templates, the FESEM image, AFM image, and TEM images of the materials confirming their sheet‐like structure and the existence of meso and macroporosity in their structure, Reproduced with permission^[^ ^131^ ^]^ Copyright Royal Society of Chemistry 2018, f) synthesis schematic of rice husk and NaCl and KCl templating based porous carbons, Reproduced with permission^[^ ^132^ ^]^ Copyright Elsevier 2021, g) equilibrium binary diagram of NaCl‐KCl salt mixture, Reproduced with permission^[^ ^133^ ^]^ Copyright MDPI 2025, h) synthesis schematic of lotus root and NaCl and KCl templating based porous carbons, Reproduced with permission^[^ ^134^ ^]^ Copyright Springer 2022, and i) KCl and MgO‐based dual salt templating to produce porous carbons from polyethylene glycol for energy storage, Reproduced with permission^[^ ^137^ ^]^ Copyright Wiley 2023.

NaCl and KCl eutectic mixture‐based templating is the most frequently explored combination. Wang et al. (2018) employed the NaCl and KCl co‐templating effect to create porous carbon sheets from cornstalk.^[^ ^131^ ^]^ A 1:1 ratio solution of the two salts was mixed with different weight ratios of the cornstalk cores, dried in air, covered in 15 g of salt in a crucible, and then carbonized at 800 °C in a muffle furnace before being washed with 1M HCl to yield the final product (**Figure** **14**a). This strategy prevents excessive biomass burning by covering the salt, which seals it from the temperature effect at < 669 °C, while the liquid‐phase salt infiltrates the carbon matrix beyond 669 °C to etch the carbon structure, forming micro, meso, and macropores. Washing the salt leaves behind 10 nm‐thick, porous carbon sheets, as observed by SEM (Figure 14b), and 4.6 nm‐thick sheets by atomic force microscopy (AFM) (Figure 14c). TEM imaging confirmed a majority of macropores with 50–200 nm size domains and mesopores of size 2–7 nm (Figure 14d and e). Although the surface area of the materials was within the median range of 1588 m^2^ g^−1^, the specific capacitance values for the three and two‐electrode systems were impressive, at 407 F g^−1^/1 A g^−1^ and 413 F g^−1^/0.5 A g^−1^, respectively. This improved performance was attributed to abundant meso‐ and macropores, ultra‐thin carbon nanosheets, and a high surface area, which enabled faster ion transport and better electrode/electrolyte interactions. Salt protection phenomena were also reported by Xue et al. (2021) in their work on NaCl and KCl templating using rice husk (Figure 14f).^[^ ^132^ ^]^ At 670 °C, the double salt melted to seal the carbon from combustion, and above this temperature, the salt infused the carbon matrix, inducing the templating effect. A surface area of 977 m^2^ g^−1^, combined with a pore volume of 0.69 cm^3^ g^−1^, enabled the delivery of a specific capacitance of 288 F g^−1^ at 0.5 A g^−1^. The salt protection ability of NaCl and KCl is attributed to their forming a eutectic mixture, which lowers the mixture's melting point relative to the individual salts. This is presented as the equilibrium binary diagram of the NaCl‐KCl system in Figure 14g.^[^ ^133^ ^]^ The melting point of the mixture is 657 °C. Below this temperature and during carbonization, the salt mixture will act as a solid sealant to protect the carbon by preventing its reaction with oxygen.^[^ ^131^ ^]^ At ≈ 657 °C, depending on the ratio of NaCl to KCl used, the liquid–solid mixture will act as a further sealing agent to protect the carbon. At higher temperatures, the salt mixture would melt and seep into the carbon, etching its structure and creating porosity. Later, Lu et al. (2022) went on to build an electrochemical sensor for hesperetin by using porous carbons synthesized from NaCl and KCl templating of lotus root (Figure 14h),^[^ ^134^ ^]^ Wang et al. (2022) introduced sheet‐like morphology and mesopores in porous carbon derived from egg white using NaCl and KCl templating for supercapacitor electrodes,^[^ ^135^ ^]^ Shi et al. (2024) utilized NaCl and KCl dual salt template under freeze drying to produce porous carbon nanosheets as anodic materials for LIB,^[^ ^136^ ^]^ and Hu et al. (2023) co‐introduced MgO with KCl salt as a dual template method to devise porous carbons from polyethylene glycol for supercapacitors (Figure 14i).^[^ ^137^ ^]^ In most of these scenarios, the ratio of the salts in the mixture determines the pore architecture, materials morphology, and various other physicochemical properties. For instance, in the MgO‐KCl‐based system, MgO was kept constant, while KCl was varied to 0, 30, 50, and 70 wt%, leading to a progressive decline in pore size from 3.29 to 2.7 nm. The surface area showed a continuous increase from 613 to 1097 to 1395 m^2^ g^−1^, then dropped to 960 m^2^ g^−1^, which could have been due to pore blockage caused by excess KCl. All materials showed evidence of graphitization supported by > 1 I_G_/I_D_ ratio in Raman spectra.

Both 2D and 3D structures can be incorporated into the porous carbon derived from petroleum coke by utilizing a dual template of NaCl and NaHCO_3_ composite, as recently demonstrated by Zhang et al. (2025).^[^ ^138^ ^]^ NaCl at high temperature tends to produce 2D carbon nanosheets, and NaHCO_3_ yields porous carbons with 3D porosity. Composite salt templating performed via hydrothermal pretreatment can induce pore formation and enhance the stability of the internal pore structures. The porosity can be further improved by using chemical activation. This was demonstrated recently by Zhan et al. (2025), who produced porous carbons from poplar wood by using a LiCl/ZnCl_2_ composite salt template for an initial hydrothermal reaction at 200 °C for 10 hr, followed by chemical activation with a mixture of potassium oxalate (K_2_C_2_O_4_) and calcium carbonate (CaCO_3_) at 800 °C.^[^ ^139^ ^]^ The presence of the LiCl/ZnCl_2_ composite template during the hydrothermal reaction enhanced the structural integrity of the pore structure and eliminated any obstruction to it caused by hydrothermal reaction products. This produced a loose, unobstructed pore structure, ideally suited for further chemical activation. Zhao et al. (2025) did not resort to a hydrothermal step and instead used low‐temperature carbonization in their work on porous carbons derived from chitosan, producing materials with reasonable porosity.^[^ ^140^ ^]^ Initially, a LiCl/KCl composite salt was used to form a macroporous carbon from chitosan at 500 °C in a tube furnace, a process not possible with chitosan carbonization alone. The macroporous carbons were further transformed into high‐surface‐area (≈2400 m^2^ g^−1^), hierarchical‐porosity carbons by combined chemical activation and N‐doping with KHCO_3_ and urea at 600–800 °C. Furthermore, Yan et al. (2025) utilized LiCl and KCl composite salt to support sulfur doping in porous carbon derived from biomass.^[^ ^141^ ^]^ The template helped create a highly porous, interconnected carbon network with uniform sulfur incorporation via even dispersion of the sulfur precursor, Na_2_SO_3_. The sulfur‐doped carbon improved electrocyclic conductivity and provided more active sites, enhancing electrochemical activity. Wang et al. (2025) demonstrated that a composite salt mixture of NaCl and ZnCl_2_ was found to yield litchi peel‐derived porous carbons with optimal porosity at 700 °C, compared to 600 °C or 800 °C. The lower temperature limit (600 °C) was insufficient to achieve significant porosity, whereas the higher temperature (800 °C) led to pore collapse; consequently, the highest surface area of 1523.13 m^2^ g^−1^ was obtained at 700 °C. Overall, composite salt templating is an appealing approach for creating porous carbons with desired porosity and other physico‐chemical properties. Other dual salt mixtures, such as KCl and ZnCl_2,_^[^ ^142^, ^143^ ^]^ ZnCl_2_ and CaCl_2,_^[^ ^144^ ^]^ KCl/NaCl, KCl/LiCl and KCl/CsCl,^[^ ^145^ ^]^ KCl and MgO,^[^ ^137^ ^]^ or ternary salt composites of ZnO/MgO/CaCO_3_^[^ ^146^ ^]^ and NaCl/KCl/NaF^[^ ^147^ ^]^ have also been reported, although not frequently, like other combinations involving NaCl.

## 4 Template Recovery, Sustainability, and Scalability Considerations

The recovery and reuse of the template are feasible when inorganic metal oxides and metal salts are used to construct porous carbons. This contrasts with silica‐based hard templating, which poses serious safety, environmental, and cost challenges and is poorly recyclable. Among inorganic metal oxides, MgO can be partially recycled via acid dissolution, yielding soluble Mg^2+^, which can be precipitated as Mg(OH)_2_ or MgCO_3_. However, although promising at the laboratory scale, the recovery efficiency in large‐scale operations could be hindered by the chemical and energy costs associated with neutralization and drying. Metal oxides such as ZnO, Fe_2_O_3_/Fe_3_O_4_, and MnO_2_ require a strong acid wash, making recovery nontrivial. In comparison, NaCl and KCl offer much better sustainability, as they can be removed by water washing and recovered for reuse owing to their thermal stability and chemical inertness. This significantly reduces recovery costs and environmental impact. From a scalability perspective, NaCl, KCl, and their composites offer the highest feasibility in terms of energy efficiency, recyclability, and environmental benignity. Although not plentiful, there is sufficient evidence in the published literature on template recovery; hence, future research should focus on closed‐loop recovery and hybrid templating systems to balance the structural control of materials with sustainable recovery and ease of recovery.

## 5 Summary and Outlook

Porous carbons stand as versatile materials for applications in adsorption, gas capture, energy storage, catalysis, and various other energy and environmental fields.^[^ ^148^, ^149^, ^150^, ^151^, ^152^, ^153^ ^]^ Besides the conventional routes of chemical or physical activation and silica‐based templating to create porosity in carbon‐based materials, inorganic metal nanoparticle‐based templating is an attractive alternative that offers unique advantages from economic and environmental perspectives.^[^ ^154^ ^]^ Inorganic templates are low‐cost, versatile materials that span a wide range and provide an easy pathway to the scalable development of porous carbons with suitable characteristics. Inorganic metal oxides (e.g., MgO, ZnO, Iron oxides, and MnO_2_) generally provide structural rigidity at high carbonization temperature. They can also be used in conjunction with chemical or physical activating agents. The choice of template, based on particle size, melting point, and interaction with the carbon precursor at high carbonization temperatures, influences the pore characteristics and mechanical stability of the porous carbons. Inorganic metal oxide‐based templated porous carbons have gained significant traction in recent years. For instance, magnesium citrate (2025) can form graphitized mesoporous carbon via MgO template‐mediated synthesis,^[^ ^63^ ^]^ and open pores can be transformed into closed pores in porous carbons via MgO templating, as reported in 2024.^[^ ^64^ ^]^ Inorganic metal salts, especially the alkali metal salts (e.g., NaCl and KCl), are water‐soluble and act as inert spacers to create interconnected meso and macro networks in a porous carbon matrix. Their use can be extended to dual or ternary composites, in which the liquid phase of salts can be achieved at lower carbonization temperatures for templating. Moreover, the porous carbons synthesized by the inorganic templating method are found to be equally competitive in terms of their application attributes compared to those produced by other conventional methods. The field has flourished rapidly in the last decade or so. A diverse range of starting precursors, including biomass, polymers, resins, and others, is continuously explored using a series of inorganic templates, such as metal oxides (MgO, ZnO, Fe_2_O_3_, Fe_3_O_4_, MnO_2_), and inorganic salts (NaCl, KCl), to create porosity. While metal oxides may or may not require washing the carbonized products with dilute acid, inorganic metal salts can be effectively removed by water washing, and the salts recovered by drying. Although, from a surface area point of view, inorganic metal oxides and inorganic metal salts may produce mediocre values within a arrange of ≈500–≈1500 m^2^ g^−1^, however, their templating action, in general, generates bigger voids or spaces residing in meso and macroporous domains which makes the mass transport of gas molecules or electrolytic ions much favourable as compared to microporous materials produced by the processes such as chemical activation. This accounts for their competitive application performance as compared to porous carbons synthesized using other templating methods. Furthermore, the corrosiveness of chemical activation with KOH and ZnCl_2_, or the harshness of HF‐based template removal, can be avoided. Overall, it is a win‐win situation for the preparation of porous carbons using inorganic templates. A systematic comparison highlighting the advantages and disadvantages of the different inorganic metal oxides and metal salts is presented in **Table** **3**.

**Table 3:** Comparison of advantages and disadvantages of different metal oxide and metal salt templates for the synthesis of porous carbons.

Template	Advantages	Disadvantages	Pore size distribution	Surface area/ tap density	Device‐level implications
MgO	Good thermal stability and structural rigidity during high‐temperature carbonization. Carbon yield is high due to template inertness. Hierarchical meso‐macroporous carbons with tunable pore sizes can be produced. Removed with a dilute acid wash with a possibility of recovery and reuse.	Weak control over the development of microporosity Graphitization capability is limited, but can be improved with co‐catalysts	Micro + Mesopores, but with more of the mesopore fraction	≈1000–1400 m^2^ g^−1^ with moderate tap density of ≈0.45 g cm^−3^	Balanced ion transport and storage, good for supercapacitors and batteries, and delivers good rate performance
ZnO	Micro‐mesoporous architectures can be produced. Removed with a dilute acid wash, or Zn could be volatilized at temperatures above 900 °C to eliminate the need for an acid wash. Compatible with various carbon precursors. Can produce moderate graphitization with fine pore control	Particle reaction with carbon can reduce yield. Thermal stability is slightly lower than that of MgO. Requires precise control to avoid pore collapse.	Mainly mesopores with smaller micropores and highly open structures	≈1200–1500 m^2^ g^−1^ and a lower tap density of ≈0.35 g cm^−3^	Ideal for high‐rate supercapacitors and gas storage
Fe_2_O_3_/Fe_3_O_4_	Interconnected mesoporous and hollow structures are produced. Strong catalytic graphitization can be induced. It can be removed with an acid wash. Enhanced electrical conductivity can be produced.	A stronger acid wash can reduce yield due to carbon loss during washing.	Micropores and small mesopores	≈800–1100 m^2^ g^−1^ with a low tap density of ≈0.30 g cm^−3^	Favourable for gas capture and also for catalysis
MnO_2_	Diverse morphologies (nanorods, nanosheets, etc.) can be replicated onto porous carbons. Removal with mild acids, including oxalic acid. Promotes the formation of mesopores.	Limited catalytic graphitization ability. Lower yield and reusability compared to MgO and ZnO. Moderate thermal stability	Mainly micropores with some mesopores	≈900–1200 m^2^ g^−1^ with a moderate tap density of ≈0.42 g cm^−3^	Electrochemical energy storage and catalysis
NaCl	High thermal stability and chemical inertness. Produces a 3D macroporous structure with small mesopores. It can be removed by dilute acid or, more preferably, by water due to its high solubility. Suitable for large‐scale and green synthesis with high carbon yield.	Less effective in creating an interconnected pore network compared to metal oxides or KCl. Limited ability for catalytic graphitization.	Hierarchical macropores and mesopores of large size.	≈400–900 m^2^ g^−1^ and with a low tap density of ≈0.30 g cm^−3^	Fast mass transport, suitable for catalysis, flow reactors, or gas storage under dynamic conditions
KCl	Partial molten state templating produces pore connectivity. Promoted mesopores along with short‐range graphitization. It can be washed with dilute acid or water.	Slightly lower carbon yield than others due to partial carbon loss. Pore collapse under prolonged heating.	Hierarchical macropores along with a considerable amount of mesopores	≈400–800 m^2^ g^−1^ with a low tap density of ≈0.28 g cm‐^3^	Similar to NaCl

Despite their proven utility in creating meso‐ and macroporosity in porous carbons via templating, inorganic metal oxide‐ and salt‐based nanoparticles pose challenges for achieving a desired level of microporosity. This is often accomplished by an additional step of chemical activation of templated porous carbons by using chemicals such as KOH, K_2_CO_3_, KHCO_3,_ etc. Achieving a uniform pore size in porous carbons is, anyway, difficult due to the uncontrolled reactions that can happen during the carbonization of carbon‐containing precursors. Another aspect of inorganic templates is their recyclability and reuse, which has been demonstrated on a few occasions with remarkable results in the reproduction of porous carbons. This aspect should be encouraged more, and further studies are required to delve into it. This is an important consideration from a bulk‐synthesis perspective. Moreover, this will establish a closed‐loop recycling system for inorganic salts, which is an added benefit from a sustainability perspective. Although multi‐salt strategies have been reported, there is a lack of comprehensive studies on this topic, and exploration of different salt combinations to devise new eutectic mixtures is required, as this is likely to yield new insights in the field. Another fascinating aspect to explore could be the hybrid templating of metal oxides and metal salts to simultaneously generate micro, meso, and macropores in an interconnected fashion. Beyond conventional high‐temperature carbonization, recent optothermal manipulation techniques,^[^ ^155^ ^]^ and field‐guided stiffness control^[^ ^156^ ^]^ highlight the localized light‐induced heating effects for controlling mass transport and nanoscale assembly. Such concepts are similar to high‐temperature carbonization and activation, which could, in the future, enable better control of pore evolution in templated porous carbons.

Although there is limited information on hybrid templating, it is feasible to combine a metal oxide with a metal salt to produce porous carbons with exciting properties. In such a case, the composite template could have a water‐soluble component (NaCl, KCl) and an acid‐soluble component (MgO, ZnO, Fe_2_O_3_, Fe_2_O_3_/Fe_3_O_4_), and the pore hierarchy can be achieved through stepwise removal of the individual templates. For instance, with a KCl and MgO‐based hybrid template, KCl in the initial step can produce a sacrificial spacer effect to generate an interconnected meso macroporous framework. The MgO nanoparticles can then be removed with mild acid dissolution to create finer mesopores. Overall multimodal pore distribution can be achieved using a hybrid templating strategy. It is also possible to remove both templates with a single‐step acid wash, although a single water wash may not be sufficient to remove them and free up the pores. Both water and acid washes are spontaneous processes (ΔG° < 0) and hence thermodynamically feasible, whether carried out in a single acid wash step or in multiple steps involving separate water and acid washes. High‐temperature carbonization converts the carbon precursor into a porous carbon with a strong aromatic carbon framework, which provides robustness during acid washing. Acid washing removes template remnants from the pores and has minimal impact on the carbon structure.

**Figure 15:**
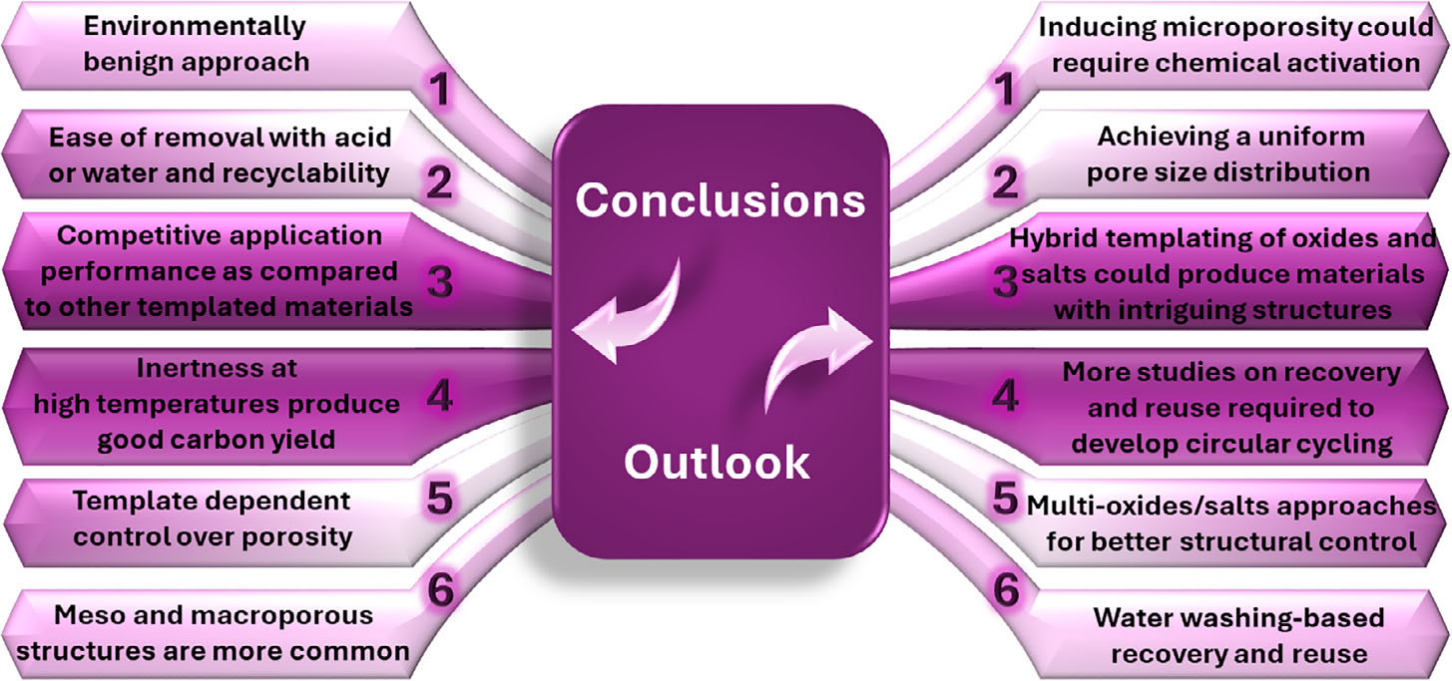
Conclusions and future outlook of the field.

Overall, the continuous evolution of low‐cost, environmentally sustainable inorganic metal oxides and alkali metal salts for templating, their advanced characterization, and their application attributes will play a key role in developing the next generation of porous carbons for various applications, including gas capture, energy storage, catalysis, and more. The key conclusions and the future outlook for porous carbons templated with inorganic metal oxides and metal salts are presented in **Figure** **15**.

## Conflict of Interest

The authors declare no conflict of interest.
